# Effects of psychedelics on neurogenesis and broader neuroplasticity: a systematic review

**DOI:** 10.1186/s10020-024-01013-4

**Published:** 2024-12-19

**Authors:** Rafael V. Lima da Cruz, Richardson N. Leão, Thiago C. Moulin

**Affiliations:** 1 Neurodynamics Lab, Brain Institute (ICe), Universidade Federal do Rio Grande do Norte, Natal, Brazil; 2 Department of Experimental Medical Science, Lund University, Lund, Sweden; 3 Department of Surgical Sciences, Uppsala University, Uppsala, Sweden

**Keywords:** Psychedelics, Hallucinogens, Neurogenesis, Dentate gyrus, Plasticity, Major depression

## Abstract

In the mammalian brain, new neurons continue to be generated throughout life in a process known as adult neurogenesis. The role of adult-generated neurons has been broadly studied across laboratories, and mounting evidence suggests a strong link to the HPA axis and concomitant dysregulations in patients diagnosed with mood disorders. Psychedelic compounds, such as phenethylamines, tryptamines, cannabinoids, and a variety of ever-growing chemical categories, have emerged as therapeutic options for neuropsychiatric disorders, while numerous reports link their effects to increased adult neurogenesis. In this systematic review, we examine studies assessing neurogenesis or other neurogenesis-associated brain plasticity after psychedelic interventions and aim to provide a comprehensive picture of how this vast category of compounds regulates the generation of new neurons. We conducted a literature search on PubMed and Science Direct databases, considering all articles published until January 31, 2023, and selected articles containing both the words “neurogenesis” and “psychedelics”. We analyzed experimental studies using either in vivo or in vitro models, employing classical or atypical psychedelics at all ontogenetic windows, as well as human studies referring to neurogenesis-associated plasticity. Our findings were divided into five main categories of psychedelics: CB1 agonists, NMDA antagonists, harmala alkaloids, tryptamines, and entactogens. We described the outcomes of neurogenesis assessments and investigated related results on the effects of psychedelics on brain plasticity and behavior within our sample. In summary, this review presents an extensive study into how different psychedelics may affect the birth of new neurons and other brain-related processes. Such knowledge may be valuable for future research on novel therapeutic strategies for neuropsychiatric disorders.

**Supplementary Information:**

The online version contains supplementary material available at 10.1186/s10020-024-01013-4.

## Introduction

According to the Global Burden of Diseases study, used by the World Health Organization (WHO) for strategic planning, 264 million people, or about 4.5% of the world population, suffer from Major Depressive disorder (MDD). It is recognized as one of the most debilitating illnesses on a global scale, substantially significantly affecting daily activities, quality of life, cognitive abilities, and work productivity (James et al. 2018). MDD is characterized by persistent anhedonia, which can be continuous or episodic, and has a profound impact on self-esteem, as well as social, family, and professional life (Lépine and Briley 2011). Mood and anxiety disorders are the most prevalent mental illnesses and the third most prevalent cause of disability, contributing to the global burden of disease (WHO 2012). The majority of pharmacological interventions aiming to treat mood disorders such as MDD are benzodiazepines or selective serotonin reuptake inhibitors (SSRIs). However, these classes of drugs do not elicit positive outcomes for about 50 to 60% of patients, leading to a condition characterized as treatment-resistant depression (TRD) (Nestler et al. 2002). SSRIs, the most modern class of antidepressants, are taken daily, with an onset of the desired effects close to one month after the beginning of treatment. However, these medications can trigger adverse effects that appear early on and last for the duration of the therapy. These drugs also have a high risk of being misused, as individuals undergoing treatment tend to become physically dependent or addicted, even with the accompanying lethargy induced by them (Wong and Licinio 2001).

The pathophysiology of depression is not yet fully understood; however, empirical data from classical antidepressants have led to the widely accepted monoamine hypothesis, which predicts that this disorder arises from a deficiency or imbalance of monoamine neurotransmitters. It is worth noting that several studies support this theory. For instance, standard antidepressants primarily operate on the monoamine neurochemical route, aiming to re-establish dopamine (DA), noradrenaline (NA), and serotonin (5-HT) levels to homeostatic concentrations. The serotonin pathway is particularly important for the monoamine hypothesis, as it is the main target of many commonly used antidepressants. There are seven main classes of serotonin receptors (5-HT1 to 5-HT7), each with multiple subtypes. These receptors are involved in a wide range of physiological functions, including mood regulation, cognition, neuroplasticity, and responses to stress and anxiety (Hannon and Hoyer 2008; Savitz et al. 2009). Importantly, Psychedelics are believed to primarily activate the 5-HT2A serotonin receptors, which are G protein-coupled receptors abundant in the cerebral cortex and are responsible for the characteristic hallucinogenic effects (Geyer et al. 2009; Nichols 2016). Activation of these receptors by substances like LSD and psilocybin leads to altered sensory perception and cognition (Carhart-Harris and Nutt 2017). The 5-HT2C receptors also contribute to the effects of psychedelics by influencing mood and anxiety regulation, although to a lesser extent (Halberstadt et al. 2011). Additionally, psychedelics may act as partial agonists at 5-HT1A receptors, affecting anxiolytic and antidepressant responses, but these play a minor role compared to 5-HT2A receptors (Nichols 2016).

Moreover, monoamine antagonists like reserpine, typically used for arterial hypertension, can induce depressive symptoms when taken over extended periods (Baumeister et al. 2003; Freis 1954; De Freitas et al. 2016); Third, treatments for MDD and anxiety disorders usually require chronic, daily dosages for at least a month to produce meaningful effects (Kempermann 2002). The latter observation has also led to a reinterpretation of the long-standing monoamine hypothesis of depression to what is now termed the neurogenic hypothesis of depression. This revised theory suggests that depression correlates with a decrease in the formation of new neurons in the adult brain, a process that seems to be revived by prolonged antidepressant treatment (Jacobs et al. 2000).

Adult neurogenesis is the process by which new neurons are generated within specific brain niches throughout the life of an organism. Neurogenesis seems to be ubiquitous to all species with a central nervous system (CNS) (Barker et al. 2011), and for many of them, the process is confined to specific regions (Barnea and Pravosudov 2011; Drew et al. 2013). In rodents, it is restricted to two zones: the olfactory bulb (OB), driven by the neural stem cells (NSCs) located in the subventricular zone (SVZ), and the dentate gyrus sub-region of the hippocampus, driven by the radial glial-like cells (RGL) (Laplagne et al. 2006). The foundations of the neurogenic theory of depression are supported by empirical data from clinical and preclinical studies aimed at understanding how the neurogenesis process is reverted to homeostatic levels when SSRI chronic treatment is applied (Miller and Hen 2015). However promising, alternative pathways to the proposed hypothesis are under discussion (Data-Franco et al. 2017; N. X. Li et al. 2022; Raphael Mechoulam and Parker 2013; Sanches et al. 2021; Yuan et al. 2015) and new biochemical routes to treat depression are emerging, including the induction of neurogenesis independent of direct 5-HT modulation (Idell et al. 2017; Reiche et al. 2018). Among the chemical candidates for novel antidepressants, encouraging results have been found with the use of psychedelics (Aleksandrova and Phillips 2021; DeVos and Miller 2013; Muttoni et al. 2019).

Psychedelics are shown to induce a range of effects on brain plasticity by changing neuronal functionality at the molecular level and producing electrophysiological changes that stimulate neurotrophic signaling, including of Brain-Derived Neurotrophic Factor (BDNF), a key promoter of synaptic plasticity and neuronal survival (Browne and Lucki 2013; Castrén et al. 2007; Magaraggia et al. 2021; Muscat et al. 2021). Ultimately, neurotrophic factors induce neurite growth (Numakawa et al. 2010; Saengsawang and Rasenick 2016; Thompson et al. 2012), synaptic remodeling (Liu et al. 2013; Zhou and Song 2001), neurogenesis (García-Cabrerizo and García-Fuster 2016; Lima da Cruz et al. 2018; Liu et al. 2017), and oxidative stress reduction (Frecska et al. 2013, 2016; Szabo 2015). Thus, it is believed that psychedelics can create a window of opportunity for therapists to introduce cognitive-behavioral treatment strategies and produce long-lasting effects, which are independent of the classical pharmacological approaches to treat the hypothesized neurotransmitter imbalance (Keeler et al. 2021; Nichols 2016; Worrell and Gould 2021). Such a holistic and personalized approach can better integrate patients into the treatment process, reducing the current disconnection between popular beliefs on mental illnesses and scientific-guided psychiatric interventions (Healy 2004; Lacasse and Leo 2005).

Despite the encouraging perspectives on the applications of psychedelics, their safe employment requires a deeper understanding of their mechanisms, as the currently available compounds generally target multiple neurotransmitter systems and may lead to undesired effects (Belouin and Henningfield 2018; Brunton et al. 2011; Geyer et al. 2009). Moreover, the effects on brain physiology are shown to depend on ontogeny (Liu et al. 2006; Riga et al. 2016; Skaper and Di Marzo 2012), gender (Lee et al. 2014; Realini et al. 2011; Rubino et al. 2008), dose (Fortunato et al. 2009; Maeda et al. 2007; Marinova et al. 2017) and chemical interactions (Canales and Ferrer-Donato 2014; Zuo et al. 2018). For this reason, we sought to cover the effects of such compounds on the plasticity process associated with neurogenesis in the dentate gyrus (DG), rather than in the SVZ-OB system (Christie and Cameron 2006; Kempermann 2012). To categorize these compounds, we adapted a classification done elsewhere (Calvey and Howells 2018). Finally, we discuss findings encompassing any effect on the molecular, cellular, physiological and behavioural levels reported for in vivo or in vitro models related to neurogenesis.

## Methods

### Search and inclusion criteria

We followed the Preferred Reporting Items for Systematic Reviews and Meta-analysis (PRISMA) guidelines to standardize the workflow (Liberati et al. 2009). The literature search was conducted in January 2023 using the PubMed and ScienceDirect databases, combining the keywords “psychedelics” and “neurogenesis” along with their respective MeSH terms. No restrictions were placed on the publication date to ensure a comprehensive assessment of the available research. Titles and abstracts were initially scanned to identify articles presenting original data related to the effects of psychedelic compounds on neurogenesis or related plasticity processes. For the purpose of this review, neurogenesis was defined as the complete process of generating new neurons through cell division, encompassing several stages: proliferation (the multiplication of neural stem or progenitor cells), differentiation (commitment of cells into specific neuronal lineages), migration (movement to designated locations), maturation (development of dendrites, axons, and synaptic capabilities), integration (incorporation into existing neural networks), and survival (the persistence of these neurons within the neural circuitry). We included studies that addressed any or all of these stages to capture the full scope of how psychedelics might influence neurogenesis and broader plasticity processes.

**Fig. 1:**
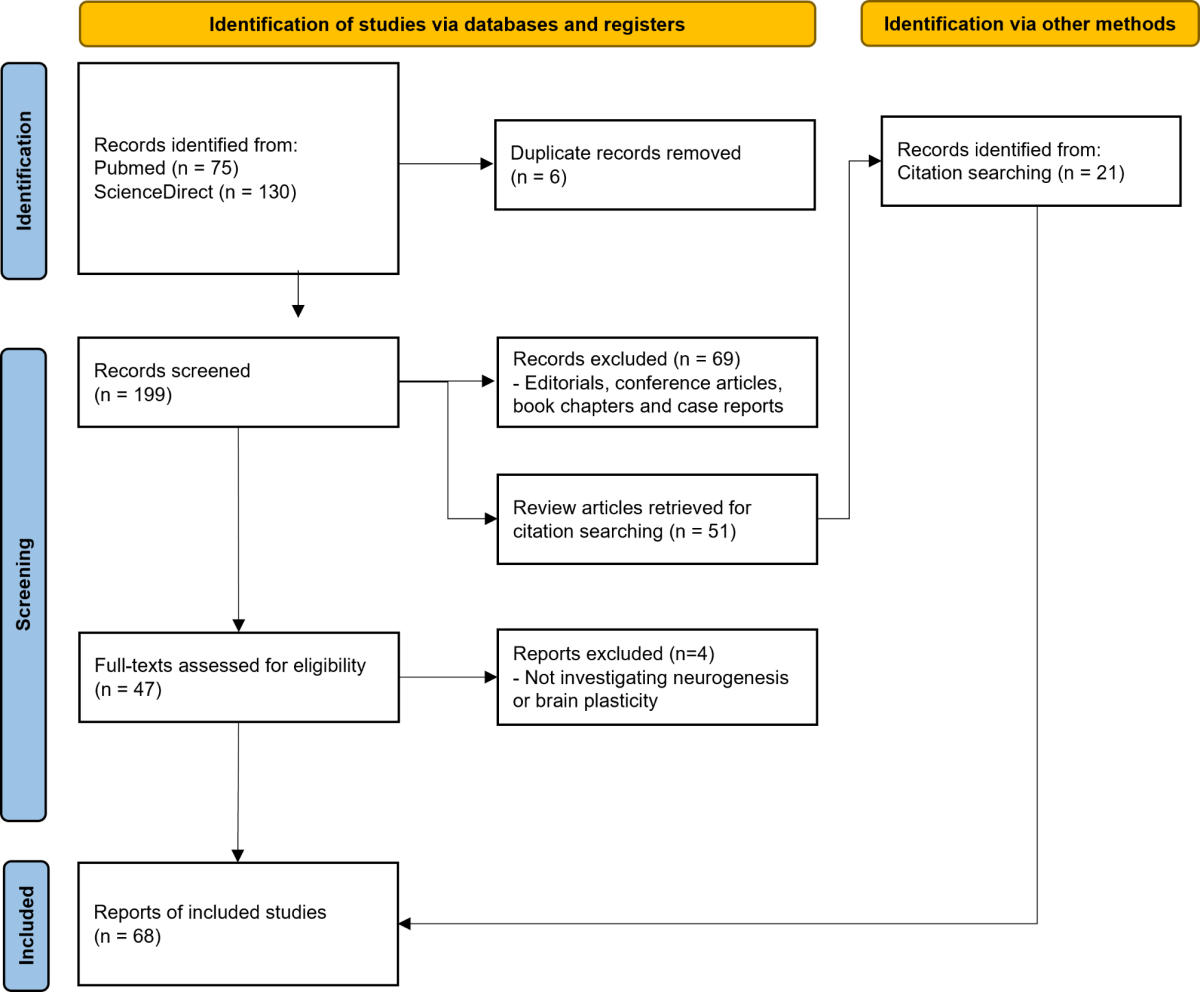
Flowchart of article selection following PRISMA guidelines. A total of 205 articles were initially identified from PubMed and Science Direct databases. After assessing abstracts, and conducting full-text analysis, 68 experimental articles were included in the final sample

Psychedelics were defined and classified as described in (Calvey and Howells 2018). Articles written in languages other than English were considered during the initial search phase, though none ultimately met our inclusion criteria, leading to a final selection of English-language studies. After the systematic search, we conducted a reference screening of all related reviews to ensure the completeness of our dataset. Experimental articles with titles mentioning any psychedelic drug were selected for abstract analysis under the same inclusion criteria. We excluded duplicates, conference notes, editorials, book chapters, and case reports during this screening process. Additionally, articles that did not assess psychedelics in any organic model or failed to discuss any effect on neurogenesis were removed. In the abstract phase, we also included articles that reported articles that used neuroplasticity biomarkers as an outcome measure were included even if they did not directly assess neurogenesis or cell proliferation. This was done to encompass a broader set of studies related to neurogenesis-associated plasticity, recognizing the limitations of direct neurogenesis measurement, especially in human studies. Following abstract screening, selected articles were sorted by the chemical group of the psychedelic for further scrutiny. Although review articles were not included in the quantitative analysis, they were considered during the discussion phase to provide additional context. The entire article selection process is illustrated in the PRISMA flowchart shown in Fig. 1.

### Data extraction and analysis

**Fig. 2:**
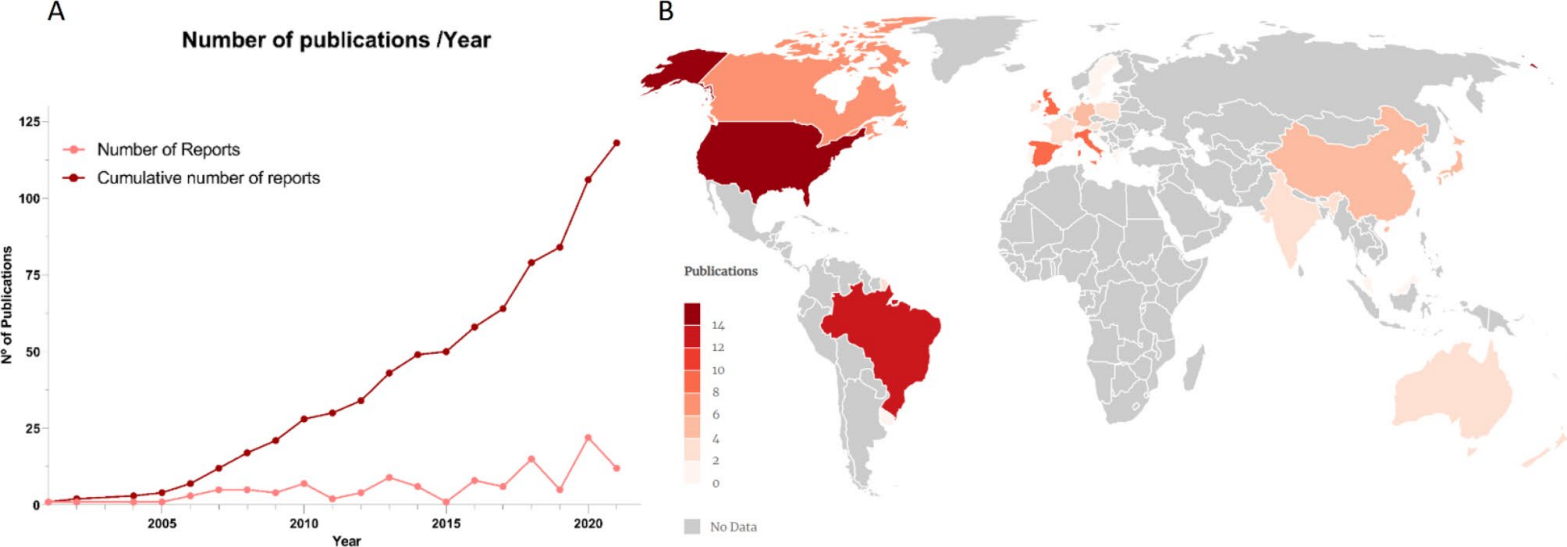
(**A**) Publication trend as cumulative (dark red) or absolute (light red) number for original articles and reviews identified by our search. (**B**) Localization of laboratories, identified by the affiliation of the correspondent author, which published original articles investigating the relationship between psychedelics and neurogenesis or neurogenesis-related plasticity (darker colours represent a higher number of publications, grey locations denote no publication record in our sample)

Data handling was performed as previous systematic reviews from our group (Moulin et al. 2020, 2022). Briefly, articles were organized in an Excel file by (1) article information, containing first and last author, author affiliation, journal, title, year of publication, and psychedelic chemical group; (2) methods information, such as drug dose and concentration, treatment regimen, experimental model of choice, techniques used, and (3) summary of results, highlighting outcome variables and reported effect on neurogenesis. For examination, a study location map (Fig. 2) was generated using a web tool (www.mapinseconds.com), and an analysis of publications over the years (Fig. 2) was performed using Microsoft Office Excel and GraphPad Prism.

### Methodological quality assessment

**Fig. 3:**
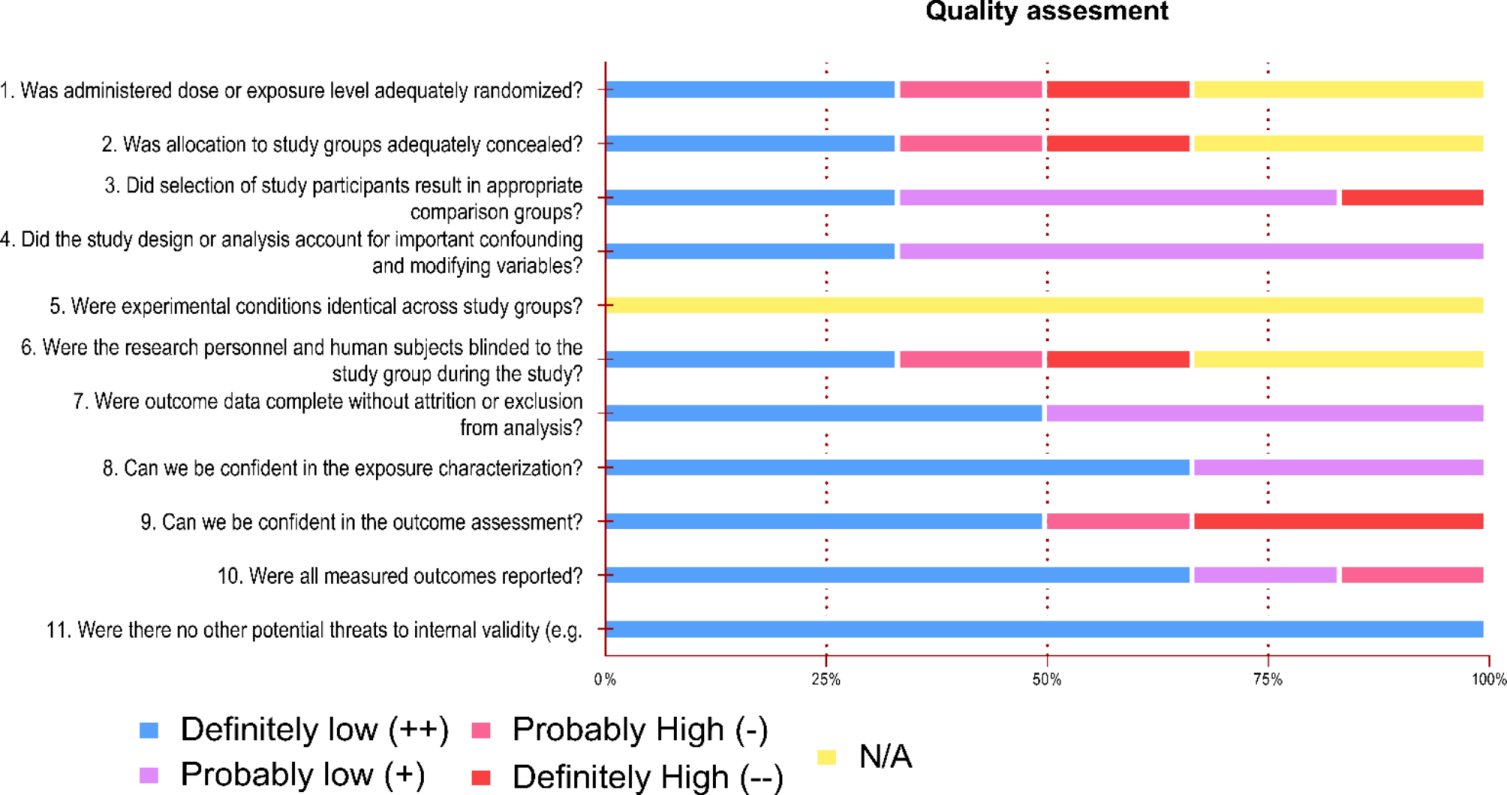
Results of OHAT Risk of Bias quality assessment of studies using human subjects. Percentages of studies within a risk of bias level are represented in the x-axis for each risk of bias item. Low risk of bias (blue, purple), unclear risk of bias (yellow), and high risk of bias (pink, red)

**Fig. 4:**
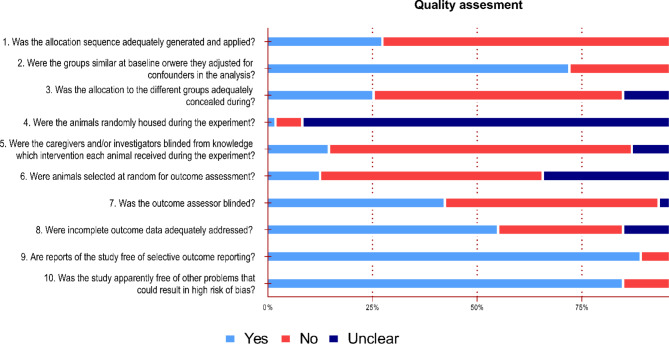
Results of SYRCLE Risk of Bias Quality assessment for the studies with animal experiments. Percentages of studies within a risk of bias assessment (Yes/No/Unclear) are represented in the x-axis for each risk of bias item

We performed a qualitative analysis of the in vivo, animal and human studies, applying the criteria from SYRCLE (Hooijmans et al. 2014) and OHAT (OHAT 2015) risk of bias (RoB) assessment tools, respectively. Both tools focus on methodological application, randomization of subjects, experimenter blinding, outcome measurements and reporting of methods. The results are reported in Fig. 3 for human studies and Fig. 4 for in vivo studies. Excel tables were generated for all categories, summarizing the method and results. In vitro studies were not analyzed for risk of bias. Considering the purpose of this review, electrophysiological assessment of acute slices was considered ex vivo and thus analyzed with the animal studies via SYRCLE RoB. However, long-term cultured neurons, even when removed from embryos, were not considered ex vivo but in vitro. Articles containing in vitro and in vivo experiments report their results independently. Finally, for the first criterion of SYRCLE RoB, “*Was the allocation sequence adequately generated and applied?*”, due to the poor reporting in this category, we graded positively articles that described any level of randomization. Moreover, for the second criterion of the SYRCLE RoB “*Were the groups similar at baseline*,* or were they adjusted for confounders in the analysis?”* for a study to be classified positively, we deemed necessary the reporting of age, gender and, when applicable, disease model onset.

## Results and discussion

### Article search and inclusion

For this systematic review, we followed the PRISMA recommendations (Liberati et al. 2009), and included articles from two databases (PubMed and Science Direct) to identify articles related to the effects of psychedelics on neurogenesis and neurogenesis-related neuronal plasticity. A total of 205 articles were initially identified, 75 from PubMed and 130 from Science Direct. First, we assessed the abstracts of selected articles, removed conference notes, editorials, book chapters, and case reports (*n* = 69), and excluded 32 publications that did not test or discuss the effects of psychedelics on neurogenesis or neuronal plasticity. Four additional articles were excluded after a full-text analysis for the same reasons. Lastly, narrative reviews were screened for further article selection, following the abovementioned criteria, resulting in 21 additional references. Our final sample contained 68 experimental articles examining the effects of psychedelics on neurogenesis or related brain plasticity outcomes. Of these, 6 were conducted in humans, 44 in vivo, and 11 in vitro. Additionally, 7 of the studies used both in *vivo* and in *vitro* approaches. Following article inclusion, we extracted metadata such as location, year of publication, and authorship, in addition to the methodological information and summarization of results. Figure 1 summarizes the searching and selection process.

### Publication trends

The oldest articles in our sample date back to 2001, indicating that investigations on the effects of psychedelics on brain plasticity are a rather recent pursuit of the neuroscientific field. Adult neurogenesis, the process by which new neurons are continuously added to the brain, was first evidenced by Altman and Das in 1965 through autoradiographic and histological evidence of postnatal hippocampal neurogenesis in rats (Altman and Das 1965). Altman further showed continuous neurogenesis in the anterior forebrain, specifically in the olfactory bulb, in 1969 (Altman 1969). Kaplan and Hinds later confirmed these findings in 1977 using electron microscopy, and identified new neurons in the rat dentate gyrus (Kaplan and Hinds 1977). Despite these findings in rodents, neurogenesis in adult humans was not widely accepted until 1998 (Eriksson et al. 1998) – although still controversial for part of the neuroscience community (Sorrells et al. 2018).

Moreover, the prohibition of psychedelics by the UN Act of 1971 may have contributed to delaying research on these compounds (Ninnemann et al. 2012; Rucker et al. 2018). Nevertheless, it is worth highlighting that attention towards psychedelics has experienced a substantial increase during the past two decades (Fig. 2A), with the most prolific publication years being 2013 (8 original articles; 2 reviews), 2018 (9 experimental articles; 6 reviews), and 2020 (8 experimental articles; 14 reviews). In 2021, a reduction in the number of published articles can be observed, likely a residual effect of the COVID-19 pandemic, which hindered lab work, funding, and collaborative research worldwide. This pause may reflect temporary logistical challenges rather than a waning interest in the field.

Lastly, we can observe that most of the publications in our sample originated from a limited number of countries (Fig. 2B), most of which have a well-documented history of high scientific productivity (King 2004). The USA produced the highest number of studies (*n* = 18), which primarily focused on the effects of ketamine (*n* = 7) and cannabinoids (*n* = 4). Notably, Brazil deviates from this trend as the only emerging economy displaying a substantial volume of publications in the field of psychedelic research. This may be explained by the legal status of the consumption of the *ayahuasca* brew, allowed for religious practices (Labate and Feeney 2012; Svobodny 2014), as it is a traditional drink consumed ritualistically by indigenous people in the Amazonian basin (Labate and Cavnar 2013). Accordingly, most Brazilian articles reviewed investigate the *ayahuasca* concoction or its main components, such as β-carbolines and tryptamines (*n* = 8).

### Sample description and risk of bias assessment

A summary of methodological descriptions from the studies can be found in Supp. Tables 1, 2, 3, 4, 5 and 6. The main findings and discussion points of each of these studies are outlined in Tables 1, 2, 3, 4, 5 and 6 and the following sections of this article.

#### Human studies

Of the 68 experimental articles identified in this study, 6 involved human participants. One case-control study examined the balance of CBD and THC phytocannabinoids in hair samples and their relationship to hippocampal mass (Demirakca et al. 2011). The other five studies explored the effects of ketamine in humans, including one cross-sectional study conducted with ketamine-addicted patients (Fan et al. 2015), and four controlled trials (Dakwar et al. 2018; Duncan et al. 2013; Haile et al. 2014; Rybakowski et al. 2013). Quality assessment using the OHAT risk of bias tool is presented in Fig. 3. Overall, the majority of articles showed ‘definitely low’ or ‘probably low’ risk of bias for most items. However, several articles had higher levels of bias or incomplete information for group randomization, allocation of subjects, and blinding. The criteria 5 is not applicable (NA) to the human studies included in our review. Moreover, half of the studies had subpar levels of outcome assessment confidence.

These results point to a diverse range of methodological robustness within the reviewed literature. The prevalence of low risk of bias in most articles suggests adherence to good research practices, implying a reliable foundation for drawing meaningful conclusions. Nonetheless, the identification of higher levels of bias in some studies and incomplete information in group randomization, allocation of subjects, and blinding, highlights specific areas for improvement in future research.

#### In vitro studies

Of the 18 original articles performing in vitro experiments, the majority utilized cannabinoid agonists (52.6%) and tryptamines (21%). The in vitro model most used was cultured cortical neurons extracted from rodents at different stages of embryonic development (26%). Risk of bias quality assessment was not performed for in vitro reports.

#### In vivo studies

In our sample, 51 original articles reported in vivo experiments in animal models. The most commonly used psychedelics administered were cannabinoid agonists (29.4%), NMDA antagonists (27.4%), and tryptamines (17.6%); however, a wide range of drugs and dosages were used in these studies. The animal models most present were rats (66.6%), followed by mice (31.4%). Other models, such as chick embryos, *Xenopus* frogs, and *Drosophila* larvae, were present in one study each.

We performed a quality of report analysis using the SYRCLE Risk of Bias tool (Hooijmans et al. 2014), and results are summarized in Fig. 4. Nearly all in vivo studies did not report a valid method of randomization, so we reduced the quality level of the report as recommended by the SYRCLE initiative. We graded these studies as “Yes” if the authors mentioned any method of randomization, regardless of its robustness. The second major source of bias in these studies was the lack of control over housing conditions, as only one article described the housing of experimental animals during the experiment in a satisfactory manner. In addition, for criteria 7 (40%), 3 (24%), 5 (14%), and 6 (12%), the number of studies graded positively did not reach 50% of the total pool, indicating a lack of rigor in most in vivo studies, which is a common issue not only in psychedelic science but across a range of experimental and methodological articles within the life sciences (Carneiro et al. 2018; Macleod et al. 2015; Moulin et al. 2020). These results highlight the importance of improving quality of reporting for increasing reproducibility in biological research (Begley and Ioannidis 2015; Ioannidis 2014). In the following sections, we will delve into each chemical category in more depth, with percentages in parentheses indicating the proportion of the total number of articles (*n* = 68) unless otherwise stated.

### Summary of findings

#### CB1 agonists

Cannabinoids are unique in the CNS. The CB1 receptor, first cloned in Tom Bonner’s lab (Matsuda et al. 1990), is predominantly expressed in neurons and is responsible for the psychoactive effects of cannabinoids (Raphael Mechoulam and Parker 2013). The CB1 receptor remained an “endogenous orphan” until 1999, when Mechoulam’s team discovered the arachidonoylethanolamide (AEA) and the 2-arachidonoylglycerol (2-AG) (Martin et al. 1999). In contrast, the CB2 receptor is less prevalent in the brain but is widely expressed in the immune system (Onaivi 2006; Pertwee 2006). While observational studies of Cannabis users, who consume the phytocannabinoids THC and CBD, have enhanced our understanding of cannabinoid effects on humans (Bonini et al. 2018; Chang and Chronicle 2007), mechanisms underlying their homeostatic functions were only recently elucidated (de Fonseca et al. 2005).

The endocannabinoid system is a complex and widespread system that acts retrogradely as a feedback channel, influencing the release of all classical neurotransmitters (Skaper and Di Marzo 2012), while enabling short- and long-term adaptive responses that lead to plasticity changes through mechanisms yet not fully understood (Araque et al. 2017). While not all cannabinoids are psychoactive or considered psychedelics (Bonini et al. 2018), we reviewed CB1 agonists due to their growing medical use and the unexplored variability in potency and effects of their synthetic analogues.

We identified 21 in vitro and in vivo studies examining the effects of five cannabinoids: synthetic compounds HU210 (23.8%), Win55212-2 (9.5%), and O-2545 (4.7%); the endocannabinoid AEA (14.2%); and THC (61.9%). Only one report included humans—a case-control study (Demirakca et al. 2011). Most experiments were conducted in vivo (71.4%), focusing on adolescent murine models (47%) to study Cannabis misuse, the most common illicit drug globally, particularly among youth (Peacock et al. 2018). All studies used molecular or cellular measurement methods, with less emphasis on behavioral (38%) and electrophysiological (9.5%) measures. Descriptive results are summarized in Supp Table 1.

The relationship between THC and neurogenesis, as detailed in Table 1, appears negative but is conflicting among different study designs. THC dosages vary greatly, with some studies administering high doses up to 30 mg/kg, which are not considered therapeutic in humans and may be excessive even for individuals with high tolerance (Downer et al. 2007; Kochman et al. 2006); Karila et al. 2014). Conversely, other studies have examined a wide range of doses from 0.75 to 10 mg/kg (Beiersdorf et al. 2020; Cuccurazzu et al. 2018; Downer et al. 2007; Kochman et al. 2006; Leishman et al. 2018; Poulia et al. 2021; Realini et al. 2011; Rubino et al. 2008; Steel et al. 2014; Suliman et al. 2018). The intervention periods also diverged significantly, including embryonic/early post-natal exposure (Beiersdorf et al. 2020; Berghuis et al. 2007; Downer et al. 2007; Psychoyos et al. 2008); adolescence in murine models, especially rats between P28–P45 (Cuccurazzu et al. 2018; Lee et al. 2014; Poulia et al. 2021; Rubino et al. 2008; Steel et al. 2014; Suliman et al. 2018); adulthood (Jiang et al. 2005; Kochman et al. 2006; Leishman et al. 2018; Rueda et al. 2002); and in vitro studies with various models (Shum et al. 2020; Stanslowsky et al. 2017; Zhang et al. 2009; Zhou and Song 2001).

Multiple studies reported a negative or negligible impact of acute or chronic CB1 agonists on neurogenesis (Berghuis et al. 2007; Downer et al. 2007; Kochman et al. 2006; Stanslowsky et al. 2017; Steel et al. 2014). Most studies focused not directly on neurogenesis but on other indirect plasticity markers (Berghuis et al. 2007; Poulia et al. 2021; Stanslowsky et al. 2017) or teratogenic morphological effects (Beiersdorf et al. 2020; Downer et al. 2007; Psychoyos et al. 2008). However, three studies identified positive effects on adult plasticity, specifically on the survival of newborn neurons and neurite growth, both in vivo and in culture (Jiang et al. 2005; Suliman et al. 2018; Zhang et al. 2009).

Several studies offer unique insights into the complex effects of cannabinoids on neural functioning and development. Parolaro’s group employed a chronic, escalating THC regimen over several days, administered twice daily across three age windows (2.5 mg/kg during P35–P37; 5 mg/kg during P38–P41; 10 mg/kg during P42–P45). Their findings highlighted three significant outcomes: first, chronic THC exposure during adolescence reduced BDNF levels via the CREB pathway in the hippocampus and prefrontal cortex of males, while paradoxically increasing BDNF in the nucleus accumbens of females (Rubino et al. 2008). Second, they observed a reduction in cell proliferation in females, an effect prevented by the FAAH inhibitor URB597 (Realini et al. 2011). Lastly, they demonstrated that this THC exposure regimen decreased both proliferation and survival rates of adult-born granule cells in female rats (Cuccurazzu et al. 2018). Expanding on these findings, Antoniou’s group showed that chronic THC exposure during adolescence disrupts dopaminergic transmission in several brain regions, providing a broader understanding of the systemic consequences of cannabis abuse during this critical developmental period (Poulia et al. 2021). Similarly, Lee et al. utilized a chronic, escalating exposure to the synthetic cannabinoid HU210 during adolescence (0.025–0.050–0.100 mg/kg) and discovered reduced proliferation and survival rates, but notably only in male mice evaluated at P70 (Lee et al. 2014).

Moreover, in a study involving a transgenic Alzheimer’s disease (AD) model, Chen et al. administered a daily dose of HU210 (0.010 mg/kg) chronically for 10 and 20 days to both old and adolescent mice. They observed a reduction in BrdU + cell numbers in young mice, and notably, most animals did not survive the planned 20-day treatment, leading the authors to halve the duration of treatment. This suggests the potentially harmful effects of HU210, especially given its 100–800 times higher potency compared to THC (Chen et al. 2010; Mechoulam et al. 1988). However, the study’s focus on AD-related features limits extrapolation to lower doses and healthy conditions.

Taking a different approach, Leishman and colleagues administered a single 3 mg/kg THC injection to female rats at three age windows (P35, P60, P90), followed by lipidome and transcriptome analyses two hours later. They found that endocannabinoid levels were reduced across the brain, with the greatest impact in the adult hippocampus (Leishman et al. 2018). Additionally, Beiersdorf et al. conducted proteomic analyses following prenatal THC exposure (1 or 5 mg/kg from P5–P16 or P5–P35) and reported severe deficits in mitochondrial function at P48 and P120, indicating long-term consequences from early THC exposure (Beiersdorf et al. 2020).

At the cellular level, Rueda et al. reported that a 24-hour in vitro exposure to the endocannabinoid AEA (5 µM) reduced neurite growth via ERK signaling. They also noted decreased survival rates of adult-born granule cells in adult rats following a four-day in vivo exposure to M-AEA (daily 5 mg/kg) (Rueda et al. 2002). Similarly, Zhou and Song showed that acute exposure of neuroblastoma N1E-115 cells to HU210 (0.01–100 nM) dampened neurite growth in a dose-dependent manner, mediated by intracellular cAMP (Zhou and Song 2001). Lastly, Shum et al. proposed that CB1 receptor-mediated dampening of neurite growth involves ERK and Akt signaling pathways. They demonstrated that the neurite growth ratio could be restored to control levels by the CB1 antagonist SR141716 in human-induced pluripotent stem cell-derived neurons (Shum et al. 2020).

Despite conflicting results regarding cannabinoids’ effects on neurogenesis and brain plasticity in murine models, phytocannabinoids and their synthetic counterparts have demonstrated antidepressant and anxiolytic effects in patients with major depression and those undergoing chemotherapy (Rodriguez Bambico et al. 2009). The legalization of cannabis in certain regions also provides a unique opportunity to study its effects in humans, including gender-specific consequences observed in preclinical models, as highlighted in recent studies (Matheson et al. 2020; Matteau-Pelletier et al. 2020). Moreover, it’s theorized that CB1 agonists may influence neurogenesis by modulating 5-HT neurons in the dorsal raphe nucleus and ACh neurons in the medial septum, both of which support various stages of neurogenesis (Raphael Mechoulam and Parker 2013).

Taken together, there is little preclinical evidence supporting the benefits of cannabinoids on neurogenesis, although extrapolating these results to humans warrants caution due to discrepancies in murine models and potential lack of translatability (Naqvi et al. 2019). The intricate relationship between the endocannabinoid system and other neurotransmitters, further complicated by species-specific variations, adds challenges to this research field. Given humanity’s longstanding relationship with these compounds, dating back 12,000 years (Ren et al. 2021), further exploration of their potential benefits is warranted. Importantly, considerations of their use and misuse should incorporate a global perspective, drawing on data from genetically diverse human populations worldwide (Ferber et al. 2019; Garani et al. 2021).

**Table 1:** Results reported by CB1 agonists studies

Outcome variable	Subjects	Reported results	Reference
**In vivo studies**			
Behavioural assessment (MWM; FC) Physiological assessment (Αβ deposition and β-Amyloid Precursor Protein; BRDU + cells)	Mice (P28 and P63), strain: AD model (APP23/ PS45)	- HU210 ↓ BrdU + cells in P28 mice ☆ - All other tests were comparable to controls	(Chen et al. 2010)
Behavioural assessment (FST, NSFT) Physiological assessment (BrdU+, NeuN+, TUNEL labeling)	Rats (adult males), strains: Long-Evans, Wistar, and Fischer	- Acute HU210 treatment ↑ BrdU + cells ☆ - Chronic HU210 does not change the percentage of BrdU+/NeuN cells ○ - Chronic HU210 ↓ score in FST and NSFT - Ablation of adult Hipp neurogenesis prevents HU210 effects on FST, NFST	(Jiang et al. 2005)
Behavioural assessment (Stereotypy) Physiological assessment (BrdU+, NeuN+, Cort)	Rats (P28), strain: Sprague Dawley	- HU210 ↓ BrdU+, NeuN + cells (only ♂) ○ - HU210 ↑ cortisol levels in (only ♂) - HU210 ↑ stereotypy in (only ♀)	(Lee et al. 2014)
Physiological assessment (BrdU+, NeuN+)	Rats (adults), strain: Sprague Dawley	- M-AEA ↓ BrdU + cells and NeuN + cells ○	(Rueda et al. 2002)
Physiological assessment (Lipidome and Transcriptome fold changes)	Mice (P35, P60, P90 females), strain: CD-1	- THC negatively impact genes and lipids implicated in neurogenesis in an age-dependent manner (greater effect on the adult brain) △	(Leishman et al. 2018)
Physiological assessment (JNK; Casp-3)	Rats (P4-P7, P20-P25, P90-P120 males)	- THC ↑JNK and Casp-3 activity - THC ↑ neonatal cell apoptosis ☆	(Downer et al. 2007)
Behavioural assessment (EPM; OF; FST; SPT) Physiological assessment (CB1r levels; pCREB)	Rats (P28), strain: Sprague Dawley	- THC ↑ FST score (only ♀) - THC ↑ SPT score (♂ and ♀) - THC ↓ CB1r levels in Amg, VTA and Nac (only ♀) - THC ↓ CREB activity in Hipp and PFC (only ♂) △ - THC ↑ in Nac (only ♀) - THC ↓ BDNF via CREB in Hipp and PFC (only ♂) △ - THC ↑BDNF in NAc (only ♀) △	(Rubino et al. 2008)
Behavioural assessment (FST, PFP, NOR, SIT) Physiological assessment (BRDU + cells)	Rats (P28 females), strain: Sprague Dawley	- THC ↑ FST and PFP scores - THC ↓ NOR score - THC ↓ BrdU + cells ☆	(Realini et al. 2011)
Physiological assessment (CB1r, PSD95, Syn1, Syn3, Ki67, DCX, PSA-NCAM, BrdU + cells)	Rats (P28-P42 males), strain: Sprague Dawley	- THC reverts the increase of CB1r, PSD95, Syn1, Syn3 levels induced by cognitive training △ - THC has no effect on PSA-NCAM and DCX + cells induced by cognitive training △ - THC has no effect on Hipp BrdU + cells ☆	(Steel et al. 2014)
Behavioural assessment (NOD) Physiological assessment (BrdU + cells, GFAP, DCX, Nestin, Tuj-1, BDNF)	Rats (P28 males), strain: Sprague Dawley	- THC ↑ BrdU + cells, GFAP, Nestin, DCX, Tuj-1 ☆△ - THC ↑ NOD scores	(Suliman et al. 2018)
Behavioural assessment (FST) Physiological assessment (CB1r, DCX, Neurite Growth) Electrophysiological assessment (fPSP in PFC L2→L5; fPSP in DG MPP→DG)	Rats (P28 females), strain: Sprague Dawley	- THC ↑ FST score - THC ↓ CB1r density, DCX + cells, neurite growth △ - THC ↓ fPSP amp. in PFC L2→L5 - THC ↓ fPSP slope in DG MPP→DG	(Cuccurazzu et al. 2018)
Physiological assessment (BrdU + cells)	Mice (adult males), strain: C56BL7	- THC has no effect in Hipp cell proliferation ☆	(Kochman et al. 2006)
Physiological assessment (CA1 histology, proteomics)	Mice (P5-P35 males)	- Neonatal THC exposure for 12 days reduced the number and diversity of neuronal types in Hipp CA1 of adult mice ○ - THC exposure on ages P5-P35 downregulate respiratory chain proteins in the mitochondrial complex in adulthood	(Beiersdorf et al. 2020)
Physiological assessment (BW, DA, BDNF, TrkB, SOX2, DCX, DOPAC) Behavioural assessment (OF, PPI, ASR, DT, OLT, MWM)	Rats (adult males)	- THC ↑ locomotor activity - THC did not change BW - THC did not change PPI or DT tests performance - THC reduced spatial memory efficiency on MWM but not learning of cognitive flexibility - THC ↑ CB1R expression in PFC - THC ↑ TrkB expression in Hipp △ - THC ↓ BDNF in PFC and Hipp △ - THC ↑ DOPAC/DA ratio in Hipp - THC ↓ DOPAC/DA ratio on PFC - THC ↓ DA and HVA levels on Dorsal striatum - THC ↑ DOPAC levels in the Nacc - THC ↓ DCX + and SOX2 + cells in DG △	(Poulia et al. 2021)
**In vitro studies**			
Physiological assessment (Morphology, neurite growth; Rap1 and B-Raf activation)	Cultured rat cortical neurons; HNSC.100 cells; PC12 cells	- AEA ↓ neurite growth via CB1 signalling and blocked NGF neurite growth increase in all models △ - AEA ↓ Rap1 and B-Raf activation of ERK signalling cascade through CB1R mediated effect. △	(Rueda et al. 2002)
Physiological assessment (CBR expression levels, Map2, Tuj1, Sox1, Nestin, TH, DAT mRNA levels, Methylation rate for DAT, MAP2 and CB1R genes) Electrophysiological assessment (Passive and active membrane properties, mPSC)	Small molecule neural precursor cells (smNPCs) from blood-derived induced pluripotent stem cell (hCBiPSC)	- Chronic THC and AEA treatment all doses have no effect on Tuj1, Map2, TH, DAT and CB1R mRNA levels △ - Chronic THC and AEA treatment ↓ INa inward and IK outward currents - Chronic CB treatment ↓ mEPSC frequency - Chronic AEA ↑ sEPSC frequency	(Stanslowsky et al. 2017)
Physiological assessment (CB1R expression and localization, Neurite outgrowth, Rho-A-B-C activity, ERK1 phosphorylation levels)	Cultured mouse (E18.5) GABAergic neurons; *Xenopus laevis*	- Win55212-2 induced cone growth retraction in mice cultured E13.5 GABAergic neurons, whereas CB1 antagonist induced cone growth attraction - CB1 agonist treatment change the axonal cone growth angle away from cathode in galvanotropic embryonic spinal neurons of X. laevis - CB1 and BDNF ↑ ERK phosphorylation △ - AEA induce CB1R removal from filopodia	(Berghuis et al. 2007)
Physiological assessment (proliferation, cAMP levels, PI3K/Akt and ERK activation, TuJ1 expression)	Cultured mouse (E17) NS/PCs hippocampal cells	− 48 h exposure to HU210 ↑ cultured hippocampal cell proliferation via cAMP increase and ERK signalling ○ - 8 days exposure to HU210 cells do not change TuJ1 expression patterns on cultured hippocampal cells	(Jiang et al. 2005)
Physiological assessment (CBR expression, Forskolin stimulated cAMP accumulation, morphology)	Mouse neuroblastoma N1E-115 cells	- HU210 ↑ forskolin stimulated cAMP accumulation - HU210 ↓ neurite growth in a dose-dependent manner △ - HU210 ↓ of neurite growth is mediated by cAMP increase	(Zhou and Song 2001)
Physiological assessment (Neurite growth, TRPV1 mediated Ca2 + influx)	PC12 cells	- HU210 ↑ neurite growth compared to control only when PC12 cells are raised in hyperglycemic medium △ - HU210 ↓ capsaicin Ca2 + influx	(Zhang et al. 2009)
Morphological assessment (nervous system development)	Chicken embryos (S3 ± E8)	- O-2545 gastrulation has strong dose-dependent teratogenic effects	(Psychoyos et al. 2008)
Physiological assessment (JNK, Casp-3 activation)	Cultured rat cortical neurons	- THC ↑ JNK and casp-3 activation via CB1R in a dose-dependent manner	(Downer et al. 2007)
Physiological assesment (CB1R expression and localization, Neurite outgrowth, ERK and Akt phosphorylation)	Induced pluripotent stem cells (hiPSCs)-derived cortical neurons	− 2-AG or THC for 24 h ↓ neurite outgrowth in hiPSC-derived neurons without reducing total number of branches ○ − 2-AG or THC for 30 min ↓ pERK △ - THC for 15 and 30 min ↓ pAkt △ - Control levels of pERK, pAkt and neurite growth are restored with co-administration of CB1R antagonists △	(Shum et al. 2020)
Physiological assessment (Neurite outgrowth, mitochondrial function)	Cultured mouse (E14.5) cortical cells	- THC for 24 h ↓ cellular growth of cultured cortical neurons in a dose-dependent manner ○ - THC at 7.5 µM disrupt mitochondrial membrane via CB1R activation and negatively impact mitochondrial function	(Beiersdorf et al. 2020)
**Human studies**			
Physiological assessment (CT scan of Hipp volume)	Healthy cannabis users and IQ, age- matched controls	High THC/CBD ratio levels from hair analyses correlates with decreased Hipp volume	(Demirakca et al. 2011)

☆ represents outcomes assessing cell proliferation through BrdU + labeling, ○ represents assessing neurogenesis through BrdU + NeuN + cell co-labeling or histological neuronal counting, and △ indicates outcomes assessing cellular processes related to proliferation, differentiation, or progenitor cell maintenance. Abbreviations: MWM (Morris Water Maze); FC (Fear Conditioning); BrdU (Bromodeoxyuridine); FST (forced swim test); NFST (novelty suppressed feeding test); NOD (novel object discrimination); cort (corticosterone); NeuN (hexaribonucleotide binding Protein-3); DCX (doublecortin); Tuj-1 (neuron-specific class III beta-tubulin); M-AEA (Methanamide); JNK (c-Jun N-terminal kinases); Casp-3 (caspase 3); fPSP (field post-synaptic potential); mEPSC (miniature spontaneous excitatory post synaptic currents); DG (Dentate Gyrus); VTA (Ventral Tegmental Area); Hipp (Hippocampus); MPP (medial perforant path); Rap1 (ras-proximate-1 or ras-related protein 1); B-raf (serine/threonine-protein kinase B-Raf); ERK (extracellular signal-regulated kinase); PPI (pre-pulse inhibition); ASR(Acoustic Startle reflex); DT (discrimination tests); OLT(object location test); smNPCs (small molecule neuronal precursor cells); Map2 (Microtubule-associated protein 2); sox1 (SRY-box transcription factor 1); Nestin (neuroepithelial stem cell protein); TH (Tyrosine Hydroxylase); DAT (dopamine transporter); rho -A-B-C (Rho family of GTPAses ras); PSA-NCAM (polysialiated neural cell adhesion molecule); ERK1 (extracellular signal-regulated kinases); ROCK (serine-threonine kinase rho kinase); pCREB (phosphorylated cAMP response element-binding protein); syn1 (synapsin-1); syn3 (synapsin-3); PSD95 (post-synaptic density protein 95); NS/PC (neural stem progenitor cells); cAMP (cyclic Adenosine monophosphate); PI3K/Akt (phosphatidylinositol 3-kinases/Protein kinase B); N1E-115 (mouse neuroblastoma cell line); TRPV1 (transient receptor potential cation channel subfamily V member 1); PC12 (rat adrenal medulla pheochromocytoma derived cell line); hiPSC (human induced pluripotent steam cell; DAGLA (diacylglycerol lipase); FAAH (fatty acid amide hydrolase); MGLL (Monoacylglycerol lipase); CT (computerized tomography)

#### NMDA antagonists

Ketamine and phencyclidine (PCP), synthetic drugs originally discovered at the Parke-Davis Research Center, are arguably the most studied NMDA antagonists (Hoefle and Warner-Lambert 2001; Pochwat et al. 2014). Initially employed as anaesthetics in the late 20th century (Domino and Warner 2010; Journey and Bentley 2021), the compound induces a dissociative state (Domino and Warner 2010; Journey and Bentley 2021), which may lead to side effects ranging from hallucinations to mania (Javitt and Zukin 1991; Pochwat et al. 2014; Ruiz and Strain 2011). The discovery of ketamine’s rapid antidepressant effect (Berman et al. 2000) was considered by some researchers the greatest breakthrough in depression research over the past half-century (Data-Franco et al. 2017; Hashimoto 2019; Sial et al. 2020). Within our review, 20 of the included studies utilized NMDA antagonists, accounting for the second most studied chemical group of psychedelics. The time point of analysis differs between laboratories, but the single-dose paradigm, employed by 80% of ketamine studies, remains the most remarkable aspect of ketamine treatment (see descriptive results in Supp. Table 2). Ketamine’s facilitatory effect on plasticity has been thoroughly examined through physiological and behavioural measures and, to a lesser degree, electrophysiological methods (Table 2). Across diverse protocols, the focus was primarily on elucidating the cellular pathway through which ketamine increases BDNF levels, subsequently promoting synaptogenesis, neurite growth, and neurogenesis (de Vos et al. 2021). Among the in vivo ketamine studies (*n* = 10), the most frequently used behavioural assessment was the forced swim test (FST; 40%), followed by the open field test (OF; 20%). Other tests, such as the novel object recognition test (NOR), novelty suppressed feeding test (NSFT), splashing test (ST), and Morris water maze (MWM), were each utilized in 10% of the studies. Broadly, these behavioural tests aimed to evaluate cognitive abilities known to be impaired in major depressive disorders.

Ketamine has been shown to enhance synaptogenesis and neurite growth in young adult mice when administered at a subanaesthetic dose ranging from 0.25 to 10 mg/kg/day (Akinfiresoye and Tizabi 2013; Li et al. 2010; R.-J. Liu et al. 2013; Ly et al. 2018). However, three studies using doses beyond this range reported potentially harmful effects on neurogenesis and associated behaviours. First, Zuo and colleagues administered 30 mg/kg/day of ketamine to male rats between postnatal days 35–42 (P35-P42), along with 2–4 g/kg/day of alcohol over 14 days. They observed a reduction in synapsin expression in the hippocampus, a phenomenon that occurred even in the absence of alcohol. The study also reported that ketamine treatment in adolescent mice induced hyperlocomotion, anxiety (assessed using OF), ataxia, stereotyped behaviour, mitochondrial damage, and morphological abnormalities in hippocampal and cortical cells. Additionally, dopamine and glutamate levels increased in the cortex and hippocampus, suggesting a possible disruption of the CREB pathway, causing the aforementioned adverse effects (Zuo et al. 2018). Next, Huang et al. (2016) delivered four sub-anesthetic i.p. injections of 10 mg/kg ketamine over 4 h to P7 rats. This regimen led to a reduction in neuronal and glial proliferation without impacting neuronal differentiation (as assessed by B-Tubulin III/BrdU + co-labelling) up to 14 days post-injection. However, the labelled cell levels in all assays returned to control levels 21 days post-injection (Huang et al. 2016). Lastly, Schiavone et al. (2020) elucidated the mechanisms underlying the neurochemical imbalance and oxidative stress observed in adulthood following intermittent administration of 30 mg/kg ketamine to neonatal rats (P7, P9, P11). A two-fold increase in NOX2 immediately preceding the chemical insult persisted into adulthood, resulting in other observed imbalances. Although the authors did not measure the number of neural progenitors in the SGZ, it’s been found that NOX2 knockout mice exhibit a reduced number of radial glial-like (RGL) cells compared to wild-type mice in adulthood (Dickinson et al. 2011), indicating a possible bimodal, dose-dependent effect of ketamine on RGL proliferation during this specific ontogenetic window of exposure via NOX2 overexpression (Schiavone et al. 2020).

Ketamine is widely recognized for its positive influence on adult neurogenesis subprocesses, such as proliferation, differentiation, and survival of new neurons, through the upregulation of BDNF (Browne and Lucki 2013; Duncan et al. 2013; Haile et al. 2014; Keilhoff et al. 2004; Lepack et al. 2015; Rybakowski et al. 2013). However, Goulart et al. (2010) present an intriguing contrast: they observed that ketamine administration led to a dose-dependent attenuation of the BDNF increase normally stimulated by the NOR task when assessed approximately five hours post-training and injection (Goulart et al. 2010). These results may be partially explained by Huang et al. (2016), who found that high doses of ketamine administered to neonatal rats transiently reduced neuronal proliferation and altered differentiation in the DG (Huang et al. 2016). Although cell levels returned to control numbers after 21 days, cognitive deficits persisted until at least postnatal day 60, as assessed by MWM. These findings suggest that early high-dose ketamine exposure can disrupt neurogenesis during critical developmental periods, leading to lasting cognitive impairments, possibly mediated by alterations in BDNF signaling (Huang et al. 2016). It is important to note that Huang et al.‘s study involved high-dose ketamine administration in neonatal animals, differing from studies in adults where ketamine generally enhances neurogenesis and synaptic plasticity (Browne and Lucki 2013; Duman and Li 2012; Ly et al. 2018; Olson 2018). This distinction highlights the significance of dosage and developmental stage in ketamine’s effects on neurogenesis.

Further complexity is added by the findings of Petermann et al. (2021), who demonstrated that the effects of citalopram (a SSRI) on BDNF protein concentrations in the hippocampus are largely separable from its effects on neurogenesis in the dentate gyrus. Specifically, chronic citalopram treatment over 21 days enhanced adult neurogenesis in control mice without affecting BDNF protein levels. Moreover, in the absence of brain serotonin, citalopram strongly decreased BDNF concentrations while the survival of newborn cells remained unchanged (Petermann et al. 2021). This suggests that serotonin can promote neurogenesis independently of BDNF, implying that ketamine’s influence on neurogenesis may also involve modulation of serotonergic pathways.

Moreover, emerging research suggests that 2R,6R-hydroxynorketamine (2R,6R-HNK), a metabolite of ketamine, may play a significant role in mediating the observed antidepressant and neuroplastic effects. 2R,6R-HNK does not act as an NMDAR antagonist and lacks the dissociative side effects typically associated with these substances (Zanos et al. 2016). Studies have demonstrated that 2R,6R-HNK induces lasting alterations in glutamatergic synaptic plasticity within the mesolimbic circuit, affecting regions crucial for mood and reward behaviors (Yao et al. 2018). Additionally, 2R,6R-HNK has been shown to enhance structural plasticity in dopaminergic neurons through AMPAR-driven BDNF and mTOR signaling pathways (Cavalleri et al. 2018). The antidepressant actions of 2R,6R-HNK also require activity-dependent BDNF signaling to increase synaptic function in the medial prefrontal cortex (Fukumoto et al. 2019). These findings suggest that 2R,6R-HNK may contribute to the positive effects of ketamine on neurogenesis via BDNF upregulation. Additional studies on 2R,6R-HNK are crucial to clarifying the complex interactions underlying ketamine’s effects and may open promising directions for future therapeutic development.

The majority of studies on phencyclidine (PCP) have sought to elucidate the neurocognitive damage caused by chronic exposure *in utero *or neonatally. All of these studies employed physiological assessments, and nearly half of them incorporated tests for behavioural evaluation (Tanimura et al. 2009; Toriumi et al. 2012). BrdU labelling was the most frequently used method (80%). The consensus from these studies is that early exposure to PCP has a significantly detrimental impact on neurogenesis (Maeda et al. 2007; Tanimura et al. 2009; Toriumi et al. 2012) and overall plasticity (Toriumi et al. 2012; Zhang et al. 2016). In contrast, a study by Liu et al. (2006) reported different results. They administered 7.5 mg/kg PCP acutely and chronically (for 5 or 14 days) for comparison within groups. They found that acute and 5-day chronic exposure resulted in minor or no effects on cell proliferation, differentiation, and survival in the dentate gyrus of adult rats when compared to the saline control. Interestingly, the decrease induced by these treatments was restored to control levels after one week of abstinence (Liu et al. 2006). However, their study lacks detailed information on the exact age of the animals, a factor that strongly influences adult neurogenesis (Kuhn et al. 1996), only stating that it was conducted on young-adult animals, not neonatal or *in utero* subjects. In summary, it is reasonable to assert that exposure to these psychoactive substances during the early stages of neural development can induce detrimental effects. Moreover, the safety of PCP as a therapeutic agent needs further evaluation in adult models, preferably in disease-relevant models, rather than focusing solely on its teratogenic effects.

Human trials with ketamine shed light on various processes contributing to plasticity. For instance, a cross-sectional study conducted by Fan and colleagues revealed that ketamine addiction can lead to low-grade inflammation (Fan et al. 2015). Further, three other studies specifically focused on TRD patients (Duncan et al. 2013; Haile et al. 2014; Rybakowski et al. 2013). These studies found a correlation between non-responsiveness to ketamine treatment in TRD patients and decreased BDNF levels 7 and 14 days after a single 0.5 mg/kg ketamine infusion (Rybakowski et al. 2013). Interestingly, when applied in a similar protocol, this same dose led to an increase in serum BDNF levels as assessed 230 min after the infusion. This increase was accompanied by an increase in Slow Wave Sleep (SWS) duration in responders (individuals characterized by a decrease of more than 50% in their baseline Montgomery-Asberg Depression Rating Scale (MADRS) score (Duncan et al. 2013). These findings were later reproduced by a study with similar methodology (Haile et al. 2014).

Moreover, in a separate human-controlled trial at the New York State Psychiatric Institute, ketamine administered to cocaine-addicted patients who weren’t seeking treatment or abstinence, reduced their cocaine self-consumption and had high scores on the Hood Mysticism Scale (HMS) when compared to other scales like the Clinician-Administered Dissociative States Scale (CADSS) and the Near-Death Experience Scale (NDES) (Dakwar et al. 2018). These results provide some insight into the possible benefits of ketamine-induced brain plasticity and subjective psychedelic experience for addiction treatment (de Vos et al. 2021; Moulin and Schiöth 2020; Walsh et al. 2022), suggesting these aspects should be monitored in assisted psychotherapy.

**Table 2:** Results reported by the reviewed NMDA antagonists’ studies

Outcome variable	Subjects	Reported results	Reference
**In vivo studies**			
Physiological assessment (Glu, Glutamine, GABA, and PV levels; NAc DA levels and DA turnover; 8-OHdG, NOX1, NOX2)	Mice (P11 and P70 males), strain: C57BL6	- Ketamine ↑ PFC Glu and Glutamine levels (P11) - Ketamine ↑ PFC Glu and GABA levels (P70) - Ketamine ↓ PFC PV, NOX1 levels (P11 & P70) - Ketamine ↓ Nac DA levels (P11 & P70) - Ketamine ↑ NAc DA turnover (P11 & P70) - Ketamine ↑ Nac and PFC, 8-OHdG and NOX2 levels (P11 & P70) - Ketamine ↓ NAc NOX1 (P11) - Ketamine ↑ NAc NOX1 (P70)	(Schiavone et al. 2020)
Behavioral assessment (OF) Physiological assessment (Cell morphology, Syn, Glu, DA, Cit-C, Casp3 and CREB pathway protein levels)	Rats (P35-42 males), strain: Sprague Dawley	- Ketamine ↑ locomotor activity, ataxia and stereotypy - Ketamine ↑ cell morphology abnormalities in CA1, CA2 and CA3 - Ketamine ↓ Syn levels in Hipp △ - Ketamine ↑ DA, Glu, Cit-C and Casp3 levels on the Cortex and Hipp - Ketamine ↓ CREB pathway protein levels. △	(Zuo et al. 2018)
Behavioral assessment (FST) Physiological assessment (mTOR and GSK-3β activity, synaptogenesis) Electrophysiological assessment (sEPSC)	Rats (adult males), strain: Sprague Dawley	- Ketamine + lithium ↑ mTOR signaling △ - Ketamine and Ketamine + lithium ↑ sEPSC frequency and synaptogenesis; - Ketamine and Ketamine + lithium ↓ score in FST - Ketamine ↑ neurite outgrowth △	(R.-J. Liu et al. 2013)
Physiological assessment (BrdU + cells)	Rats (P56 males), strain: Sprague Dawley	- Ketamine ↑ BrdU + cells after 2 and 3 weeks ☆	(Keilhoff et al. 2004)
Behavioral assessment (NOR) Physiological assessment (BrdU + cells, BDNF levels)	Rats (P70-95 males), strain: Sprague Dawley	- Ketamine after training ↓ score in NOR - Ketamine ↓ BDNF levels increase after cognitive training in NOR △	(Goulart et al. 2010)
Behavioral assessment (FST, NSFT) Physiological assessment (mTOR signaling, Neurite outgrowth) Electrophysiological assessment (sEPSC)	Rats (adult males), strain: Sprague Dawley	- Ketamine ↑ mTOR signaling in the PFC △ - Ketamine ↑ synaptogenesis and sEPSC in PFC △ - Ketamine-induced increases in mTOR signaling and synaptogenesis is abolished by pre-treatment with NBQX or ERK blockers △ - Ketamine ↓ score in FST and NFST - Ketamine-induced reductions in FST and NFST scores are abolished by pre-treatment with rapamycin, and ERK blockers	(Li et al. 2010)
Behavioral assessment (OF, FST, SPT) Physiological assessment (BDNF, Syn and d-mTOR levels)	Rats (adult males), strain: Wistar	- Ketamine ↓ FST score - Ketamine ↑ SPT score - Ketamine ↑ BDNF, Syn and d-mTOR levels △	(Akinfiresoye and Tizabi 2013)
Behavioral assessment (FST) Physiological assessment (BDNF Levels)	Rats (adult males), strain: Sprague Dawley	- mPFC BDNF block prevent ketamine-induced FST score reduction △ - L-Type channel block prevents ketamine-induced FST score reduction	(Lepack et al. 2015)
Behavioral assessment (MWM) Physiological assessment (BrdU, Nestin, β-tubulin III, NeuN)	Rats (P7)	- Ketamine ↓ Nestin+/BrdU + cells in proliferation essay ☆ - Ketamine ↑ β-tubulin III+/BrdU + neurons in differentiation essay ○ - Ketamine ↓ astrocytes in differentiation essay - Ketamine did not change the apoptosis rate in the DG - Ketamine ↓ the migration of NeuN+/BrdU+ (red) cells ○ - Ketamine ↓ RGL growth - P7 exposure to Ketamine ↓ score in MWM when adults	(Huang et al. 2016)
Physiological assessment (Neurite outgrowth, Spine density)	Rats (P56 females), strain: Sprague Dawley	- Ketamine ↑ spinogenesis in rats Pyr PFC neurons. △	(Ly et al. 2018)
Morphological assessment (BW) Physiological assessment (PFC cell density and number, PFC cell migration, PFC, PCNA+, PAG+, Pax6+, Tbr2+, BrdU + cells, expression levels of Notch2 and Ntn1) Behavioral assessment (NOR, FST, PPI)	Mice (P1, P7 and P56 males), strain: CD-1	- PCP ↓ BW (P1) - PCP ↓ NOR and PPI score - PCP ↑ FST score; - PCP ↓ PFC cell density and PAG + cells (P56) - PCP ↓ PFC BrdU + cells (P7) ☆ - PCP ↓ PCNA+, Pax6+, Tbr2 + cells △ - PCP ↓ expression levels of Notch2 and Ntn1 △	(Toriumi et al. 2012)
Physiological assessment (BrdU + cells)	Rats (adult males), strain: Sprague Dawley	- Chronic PCP ↓ BrdU + cells ☆ - Chronic PCP did not alter survival or differentiation of abGC	(Liu et al. 2006)
Behavioral assessment (Locomotion) Physiological assessment (BrdU + cells)	Rats (P21 males), strain: Sprague Dawley	- PCP ↑ BrdU + cells ☆ - PCP ↓ locomotor activity	(Tanimura et al. 2009)
Physiological assessment (BrdU + cells)	Mice (P56 males), strain: CD-1	PCP 3, 10 mg/kg ↓ BrdU + cells ☆	(Maeda et al. 2007)
**In vitro studies**			
Physiological assessment (Neurite growth; Synaptophysin, PSD95 and BDNF levels; Akt and GSK3β phosphorylation levels; NRG1 activation levels)	Cultured mice PFC neurons	- PCP ↓ neurite growth △ - PCP ↓ Synaptophysin and PSD95 levels △ - PCP ↓ phosphorylation of Akt and GSK3β without affecting the expression levels △ - PCP reduction of neurite growth and Akt/ GSK3β phosphorylation is dependent of NRG1 activation △ - PCP ↓ BDNF levels, NRG1 activation dependent △ - PCP ↓ NRG1 expression levels △	(Zhang et al. 2016)
**Human studies**			
Survey assessment (PANSS) Physiological assessment (TNF-α, IL-6 and IL-18)	Hospitalized ketamine-addicted and matching healthy control subjects	- Ketamine-addicted subjects have ↑ IL-6, IL-8 levels and PANSS scores; - Ketamine-addicted subjects have ↓ TNF-α levels	(Fan et al. 2015)
Survey assessment (HMS, self-reports on cocaine craving and self-administration)	Cocaine-addicted subjects	- HMS correlates with overall improvement - Ketamine treatment reduced cocaine self-administration	(Dakwar et al. 2018)
Survey assessment (HDRS) Physiological assessment (Serum BDNF, NGF, NTF3, NTF4 and GDNF)	Bipolar patients within a depression episode and resistant to classical antidepressants	- BDNF levels in non-responders was reduced 7 and 14 days after treatment △ - A single dose of ketamine reduced score in HDRS in all patients 14 days after treatment - 48% of patients had a HDRS score ≤ 7 points 14 days after treatment	(Rybakowski et al. 2013)
Survey assessment (MADRS) Physiological assessment (Serum BDNF) Electrophysiological assessment (SWA)	Treatment-resistant MDD-diagnosed subjects	- Single ketamine dose ↓ MADRS score - Single ketamine dose ↑ Serum BDNF levels 230 min after △ - Single ketamine dose ↑ total sleep time, SWS and REM sleep - SWA and BDNF levels increase positively correlates in responders	(Duncan et al. 2013)
Survey assessment (MADRS) Physiological assessment (Serum BDNF)	Treatment-resistant MDD-diagnosed subjects	- Single ketamine dose ↓ MADRS score in ~ 50% of patients - BDNF increase negatively correlates with MADRS score - Single dose of ketamine ↑ BDNF serum levels △	(Haile et al. 2014)

☆ represents outcomes assessing cell proliferation through BrdU + labeling, ○ represents assessing neurogenesis through BrdU+/ (NeuN + or β-tubulin III+) cell co-labeling or histological neuronal counting, and △ indicates outcomes assessing cellular processes related to proliferation, differentiation, or progenitor cell maintenance. Abbreviations: Nac (Nucleus Accumbens); PFC (Prefrontal Cortex); glu (glutamate); DA (dopamine); PV (parvalbumin); 8-OHdG (8-Oxo-2’-deoxyguanosine); NOX1 (NADPH oxidase 1); NOX2 (NADPH oxidase 2); syn (synapsin); OF (Open Field Test); FST (forced swim test); cit-c (cytochrome complex); Casp3 (Caspase 3); Creb (cAMP response element-binding protein); mTor (mechanistic target of rapamycin); GSK-3β (glycogen synthase kinase 3 beta); sEPSC (spontaneous excitatory post-synaptic current); pyr (pyramidal neuron); abGC (adult born granule cells); GCL (Granule Cell Layer); RGL (radial-Glia like cells); NBQX (2,3-dioxo-6-nitro-7-sulfamoyl-benzo[f]quinoxaline); ERK (extracellular signal-regulated kinase); BrdU (Bromodeoxyuridine); NOR (Novel object Recognition Test); BDNF (brain derived neurotrophic factor); MWM (Morris Water Maze Test); BW (Body Weight); PCNA (proliferating cell nuclear antigen); PAG (phosphate activated glutaminase); Pax6 (paired box protein Pax-6); Tbr2 (T-box brain protein 2); Notch2 (neurogenic locus notch homolog protein 2); Ntn1 (Netrin-1); PPI (pre-pulse inhibition test); PSD95 (post-synaptic density protein 95); akt (protein kinase B); NRG1 (Neuregulin 1); PANSS (positive and negative syndrome scale); TNF-α (tumor necrosis factor-α); IL-6 (interleukin 6); IL -18 (interferon-gamma inducing factor); HMS (Hood Mysticism Scale); CADSS (Clinician Administered Dissociative States Scale); NDES (Near-Death Experience Scale); HDRS (Hamilton Depression Rating Scale); NGF (nerve growth factor); NTF3 (neurotrophin-3); NTF4 (neurotrophin-4); GDNF (glial- derived neurotrophic factor); SWA (slow-Wave activity); MADRS (Montgomery–Åsberg Depression Rating Scale)

### Harmala alkaloids

Harmala alkaloids are indole nitrogenated compounds featuring a β-carboline heterocyclic structure, first identified in the seeds of *Peganum harmala*, also known as Syrian Rue (Herraiz et al. 2010). Recently, these compounds have attracted increased attention due to their presence in *ayahuasca* brews. The primary compounds in this category include harman, harmine, harmaline, and tetrahydroharmine (de Oliveira Silveira et al. 2020). While these β-carbolines are not inherently psychedelic, they can influence serotonin signaling by inhibiting the monoamine oxidase enzyme (MAO), thus producing behavioral effects somewhat similar to SSRIs (Domínguez-Clavé et al. 2016). Often referred to as MAO inhibitors (MAOis), these compounds are ubiquitous in the various forms of *ayahuasca* prepared by indigenous populations (McKenna et al. 1984). Interestingly, in some versions of *ayahuasca*, DMT is absent (Callaway 2005; Rivier and Lindgren 1972). Their antidepressant effect has been explored in psychiatry, with some MAOis even being marketed under names like Neuralex and Marplan, among other synthetic functional analogs (Fagervall and Ross 1986; Robinson 1973). However, most MAOis were withdrawn from the market due to hepatotoxicity (Lopez-Munoz and Alamo 2009), although it may not be the case of the harmala alkaloids (Brito-da-Costa et al. 2020). The cellular mechanisms of such compounds are still being explored, more recently under the light of the neurogenic hypothesis for MDD, as their effects on neurogenesis and plasticity processes are uncovered (Ferraz et al. 2019).

All studies involving harmala alkaloids (6 included, Tables 3 and Supp. Table 3) focus on their potential antidepressant effects. In vivo studies also assessed hippocampal BDNF in conjunction with behavioral methods. Half of articles from this category originated from Quevedo’s lab, which conducted a series of rat experiments revealing the potential antidepressant effects of the harmine alkaloid (Fortunato et al. 2009, 2010a, b). The team initially found that male rats at post-natal day 60 (P60) showed improved performance in the forced swim test (a measure of learned helplessness, a common depressive-like behavior) after being treated with 10 or 15 mg/kg of harmine one hour prior to the test. Their locomotor activity (measured by open field test) remained normal. The improvement was comparable to that produced by the tricyclic antidepressant imipramine. Intriguingly, rats treated with 15 mg/kg of harmine demonstrated a two-fold increase in BDNF levels immediately after the test, while those treated with imipramine did not (Fortunato et al. 2009). The following year, the team reported similar results after chronic administration (14 days) of harmine (Fortunato et al. 2010a).

To further address the relevance of the results to depression pathophysiology, the group later generated a study incorporating a Chronic Mild Stress (CMS) model of depression in rats. There, they administered 15 mg/kg of harmine over a 7-day treatment period, using a similar protocol to their previous study, but without the imipramine control. Similar results were found. Moreover, the FST assessment was replaced with the sucrose preference test (SPT) to measure anhedonia-like phenotypes, a core symptom of depression. The study demonstrated that harmine-treated mice exhibited less anhedonia only after CMS. The study also found that harmine treatment reduced blood levels of adrenocorticotropin (ACTH), a stress hormone, while adrenal weight was similar to that of non-CMS rats. Contrary to their previous findings, BDNF levels after treatment were not affected. Such discrepancy could be due to the different time-points of sample collection employed in the study (first after a stressful situation, second after a mildly pleasant situation), suggesting that harmine may dynamically influence BDNF levels (Fortunato et al. 2010b).

Harmine was also studied by Liu and colleagues, who used Chronic Unpredictable Stress (CUS) to model depressive symptoms in mice. They examined the effects of chronic administration of 10 and 20 mg/kg of harmine using the forced swim test (FST), sucrose preference test (SPT), and tail suspension test (TST) — the three most commonly used tests for measuring depressive symptoms in mouse models. It was reported a 40% effect favoring the antidepressant action of harmine in all behavioral tests, which was comparable to the positive control, fluoxetine (Liu et al. 2017). In addition to behavioral tests, biochemical markers were investigated. The team described that 20 mg/kg of harmine increased BDNF levels in the hippocampus and prefrontal cortex, along with glutamate transporter 1 (GLT-1) expression levels. They also found that harmine promoted the survival of newborn cells, as evidenced by doublecortin (DCX) immunofluorescence, and prevented the reduction in glial fibrillary acidic protein (GFAP) induced by the CUS protocol (Liu et al. 2017). Lastly, the authors tested the hypothesis that these regulatory effects might be due to the restoration of astrocytic function by administering L-Alpha-Aminoadipic Acid (L-AAA), an astrocyte-specific gliotoxin, intracerebroventricularly (i.c.v). Indeed, this treatment shifted the results towards those of the saline-CUS group (Liu et al. 2017).

Moreover, an in-vitro study showed that the presence of 7.5 µM harmine in the culture medium of human neural progenitor cells (hNPCs) increased proliferation by 71.5% (Dakic et al. 2016). Morales-García and collaborators tested four different harmala alkaloids (harman, harmine, tetrahydroharmine, and harmol) at a 1 µM concentration, acutely (3 days) and chronically (7 days), on neurospheres derived from the subgranular zone (SGZ) and subventricular zone (SVZ) of adult mice. The treatment enhanced all stages related to neurogenesis in vitro: proliferation, migration, and differentiation (Morales-García et al. 2017). Altogether, the current body of research suggests that harmala alkaloids have a positive impact on neurogenesis and neuroplasticity, particularly within the framework of depression models (Table 3).

In summary, the antidepressant potential of MAOis is well known and has been explored in the past. However, more research is needed regarding the side effects of harmala alkaloids (Wimbiscus et al. 2010). Future studies should focus on expanding our understanding of the long-term safety of these compounds, comparing them with synthetic MAOis, preferably those still on the market. This research will help determine whether their positive effects can counterbalance potential hepatotoxicity and severe interactions with tyramine-rich foods. By identifying which part of the harmala alkaloid structure is responsible for differences in action and consequences, we can pave the way for the development of safer analogs.

**Table 3:** Results reported by the reviewed studies on Harmala alkaloids

Outcome variable	Subjects	Reported results	Reference
**In vivo studies**			
Behavioral assessment (FST; OF) Physiological assessment (Hipp BDNF)	Rats (P60), strain: Wistar	- Harmine ↓ score in FST - Harmine ↑ Hipp BDNF △	(Fortunato et al. 2009)
Behavioral assessment (FST; OF) Physiological assessment (Hipp BDNF)	Rats (P60), strain: Wistar	- Harmine ↓ FST score - Harmine ↑ Hipp BDNF △	(Fortunato et al. 2010a)
Behavioral assessment (SPT, OFT) Physiological assessment (Adrenal Weight, blood ACTH and Hipp BDNF)	Rats (P60), strain: Wistar	- Harmine ↑ SPT score - Harmine ↓ adrenal weight - Harmine ↓ blood ACTH - Harmine ↓ Hipp BDNF increase induced by CMS model △	(Fortunato et al. 2010b)
Behavioral assessment (TST; FST; SPT) Physiological assessment (Hipp and PFC BDNF; GLT-1; GFAP)	Mice (P56-P70 males), strain: C57BL6	- Harmine ↓ TST, FST scores - Harmine ↑ SPT score - Harmine ↑ BDNF, DCX + cells, GLT-1, GFAP △	(Liu et al. 2017)
**In vitro studies**			
Physiological assessment (EdU+, Nestin, GFAP cells)	Human neural progenitor cells (hNPCs)	- Harmine ↑ proliferation and neuronal differentiation	(Dakic et al. 2016)
Physiological assessment (Musashi1, Nestin, Sox-2, Tuj-1, MAP-2, CNPase, GFAP levels, Ki67 +, DCX + Cells)	Neurospheres from adult mice SGZ and SVZ	- Chronic B-Carbolines ↓ Musashi-1, Sox-2, Nestin levels in both NPC populations △ - Chronic B-Carbolines ↑ neuronal number and size of adult neurospheres in both NPC populations ☆ - Chronic B-Carbolines ↑ ki67 + cells and PCNA levels △ - Acute B-Carbolines ↑ Tuj1 and MAP-2 levels in both NPC △ populations, with exception to Harmine at SGZ for Tuj1 - Chronic Harmol and Harmaline ↑CNPase levels at SVZ population - Chronic B-Carboline ↑ GFAP in both NPC populations, with exception to Harmine at SGZ	(Morales-García et al. 2017)

☆ represents outcomes assessing cell proliferation through BrdU + labeling, ○ represents outcomes assessing neurogenesis through neuronal counting, and △ indicates outcomes assessing cellular processes related to proliferation, differentiation, or progenitor cell maintenance. Abbreviations: FST (forced swim test); OF (Open Field Test); BDNF (brain derived neurotrophic factor); SPT (sucrose preference test); TST (tail suspension test); PFC (pre frontal cortex); Hipp (Hippocampus); EdU (5-Ethynyl-2’-deoxyuridine); GFAP (glial Fibrillary Acidic protein); Nestin (neuroepithelial stem cell protein); Sox-2 (sex determining region Y-box 2); Tuj-1 (neuron-specific class III beta-tubulin); Map-2 (Microtubule-associated protein 2); CNPase (2’,3’-Cyclic-nucleotide 3’-phosphodiesterase); Ki67 (marker of Proliferation Ki-67); SVZ (Subventricular Zone); dentate gyrus of the Hippocampus (SGZ); NPC (neural progenitor cell); PCNA (proliferating cell nuclear antigen); CMS (chronic mild stress); DCX (Doublecortin); SGZ (Subgranular Zone); GLT-1 (glutamate transporter 1)

### Psychoactive tryptamines

Tryptamines are indolamines neurotransmitters that originate from tryptophan (Araújo et al. 2015). All psychoactive tryptamines are potent 5-HT2A agonists, and many also demonstrate affinity for 5-HT1 and other 5-HT2 receptors. Notably, they may also interact with ionotropic and metabotropic glutamate receptors, dopamine, acetylcholine, and trace amine-associated receptor (TAAR) (Carbonaro and Gatch 2016). Certain DMT analogs have shown activity via sigma-1 receptors as well (Fontanilla et al. 2009). This broad receptor affinity equips psychoactive tryptamines with the capability to induce altered states of consciousness, characterized by heightened introspection (de Araujo et al. 2012) and changes in sensory perception, mood, and thought (Calvey and Howells 2018).

Psychoactive tryptamines, used by ancient civilizations globally for millennia (Desmarchelier et al. 1996), were first clinically investigated in the mid-20th century for the treatment of mood and drug abuse disorders, and some advocates proposed their systematic use by healthy individuals to enhance cognition (Nichols 2016; Williams et al. 2002). Despite their shared primary pharmacologic pathway—5-HT2A receptor agonism—these tryptamines can generate different experiences due to their variable affinity for other receptors. The current review includes eleven studies that meet the inclusion criteria, investigating the effects of psilocybin (18%), ibogaine (9%), and DMT analogs, including the ayahuasca concoction (73%). These tryptamines were predominantly assessed using physiological and behavioral methodologies, with electrophysiological methods used less frequently (Supp. Table 4).

As shown in Table 4, the majority of the included studies provide evidence that, irrespective of their specific type, tryptamines have the potential to enhance aspects of neuroplasticity (Catlow et al. 2013; Dunlap et al. 2020; Katchborian-Neto et al. 2020; Lima da Cruz et al. 2018; Ly et al. 2018; Marton et al. 2019; Morales-Garcia et al. 2020). Remaining studies, although not directly assessing neurogenesis, reported no adverse effects on behavior or cellular physiology (Castro-Neto et al. 2013; Wankhar et al. 2020; Winne et al. 2020). For instance, Catlow et al. (2013) discovered that a single low dose (0.1–0.5 mg/kg) of psilocybin, administered 24 h prior to a Trace Fear Conditioning test, initially heightened fear response but facilitated fear extinction by the third trial, an effect not observed until the tenth trial in control animals. Conversely, high doses (1-1.5 mg/kg) showed no behavioral impact and reduced BrdU-labeled cells 18 days post-treatment (Catlow et al. 2013).

Wankhar et al. investigated the acute and sub-acute (7 days) effects of 0.7 mg/kg 1-Methylpsilocin, a psilocin tryptamine derivative. Their research aimed to assess the potential of 5-HT2CR exploitation in mitigating symptoms common to mood disorders. Following a Chronic Unpredictable Stress (CUS) protocol in rats, they employed OF, FST, and EPM behavioral tests in conjunction with tissue sample analysis. Their findings indicated that 1-Methylpsilocin alleviated behavioral signs of anxiety and anhedonic phenotype by the seventh day post-CUS. Furthermore, they demonstrated that this treatment could rescue subjects from CUS-induced mitochondrial damage and imbalanced 5-HT/5-HT2CR levels in the PFC and hippocampus. Remarkably, the treatment also mitigated hippocampal cell morphological abnormalities and the CUS-induced rise in corticosterone levels (Wankhar et al. 2020).

Ibogaine, a naturally occurring psychoactive tryptamine found in certain plants of the Apocynaceae family native to Central Africa, has been investigated for its potential to mitigate drug-seeking behavior (Alper et al. 2008). The compound’s influence on neuroplasticity in mesocorticolimbic areas of the rat brain has been examined, uncovering intricate interactions between NGF, GDNF, and BDNF, both in terms of mRNA and protein products (Marton et al. 2019). Briefly, 20 or 40 mg/kg doses of ibogaine (I_20_ and I_40_) dose-dependently upregulated BDNF and NGF mRNA in PFC, Ventral Tegmental Area (VTA), Substantia Nigra (SN), and Nucleus Accumbens (Nacc) 24 h post-injection. However, 3 h post-injection, a decline of 1.7 and 2-fold in PFC-BDNF mRNA was observed for the I_20_ and I_40_ groups, respectively. Notably, I_40_ also elevated GDNF levels in VTA and SN 24 h later. In terms of protein levels, upregulation was confined to proBDNF-NAcc (for both I_20_ and I_40_) and VTA-GDNF exclusively for I40, with a possible tendency for increased BDNF in NAcc and VTA that warrants further investigation (Marton et al. 2019).

The indole alkaloid 5-Methoxy-N, N-dimethyltryptamine (5-MeO-DMT), a structural analog of DMT found in the venom of the *Incilius alvarius* toad, has been the subject of several studies (Araújo et al. 2015). In a series of three experiments, our team investigated the effects of 5-MeO-DMT on adult neurogenesis (Lima da Cruz et al. 2018). Our first two experiments showed an increase in the proliferation and survival of newborn granule cells in the ventral dentate gyrus following a single intracerebroventricular injection of 100 µg of 5-MeO-DMT. In the third experiment, we employed the whole cell patch clamp technique to measure the passive and active membrane properties of the identified doublecortin-positive cells. The results revealed that the adult-born granule cells (abGCs) from treated animals exhibited electrophysiological properties more akin to mature granule cells. In a separate study, Winne et al. examined salicylate-induced anxiety-like behavior. Their findings suggested that salicylate can induce theta-2 and slow gamma oscillations in young mice, correlating with anxiogenic behavior. This effect was mitigated by a single 20 mg/kg intraperitoneal injection of 5-MeO-DMT administered a week prior to the test (Winne et al. 2020).

Moreover, a study on adult rats to assessed changes in neurotransmitter levels 40 min post-ingestion of *ayahuasca*. Their findings indicated that all tested ayahuasca concentrations (250, 500, 800 mg/kg oral) elevated GABA and 5-HT levels in the hippocampus and reduced glycine and GABA levels in the amygdala, following a dose-dependent pattern. Moreover, levels of all monoamines in the amygdala increased, yet the rate of serotonin consumption was reduced in the amygdala. Only the highest dose of 800 mg/kg could reproduce this effect in the hippocampus (Castro-Neto et al. 2013). Regarding DMT analogs, noteworthy efforts are being made by David E. Olson’s lab at UC Davis to develop psychedelics without hallucinogenic properties while maintaining their neuroplastic effects, thus potentially reducing undesirable effects for some patients. Despite certain criticisms, Dunlap and colleagues reported successful results, demonstrating that isoDMT and 5-MeO-isoDMT enhanced neurite growth in cultured cortical neurons, much like their natural counterparts. Interestingly, in further experiments, these compounds did not elicit the head-twitch response (HTR) - a behavior commonly observed in mice when a hallucinogenic 5-HT2A agonist is administered (Dunlap et al. 2020).

**Table 4:** Results reported by the reviewed studies on psychoactive tryptamines

Outcome variable	Subjects	Reported results	Reference
**In vivo studies**			
Physiological assessment (Neurite outgrowth, Spine density) Electrophysiological assessment (sEPSC)	Rats (P56 females), strain: Sprague-Dawley; *Drosophila melanogaster* adults	- DOI, LSD ↑ Neurite outgrowth in *Drosophila* and Rats △ - DMT and ketamine ↑ spine density PFC Pyr neurons △ - DMT ↑ sEPSC frequency and amplitude in PFC Pyr neurons	(Ly et al. 2018)
Physiological assessment (BrdU + cells, DCX + cells, Neurite outgrowth) Electrophysiological assessment (Passive and active membrane properties)	Mice (P55-P70 males), strain: C57BL6	− 5-MeO-DMT ↑ BrdU + cells in vDG ☆ - 5-MeO-DMT ↑ DCX + cells in vDG △ - 5-MeO-DMT ↓ AHP and AP threshold in young GC on vDG - 5-MeO-DMT ↑ maturation rate of young GC on vDG △ - 5-MeO-DMT ↑ number of branches of young GC at vDG △	(Lima da Cruz et al. 2018)
Behavioral assessment (OF) Electrophysiological assessment (LFP)	Mice (P120-P150 males), strain: C57BL6	− 5-MeO-DMT ↓ anxiety-like behavior and electrophysiological LFP signature of anxiety	(Winne et al. 2020)
Behavioral assessment (OF) Physiological assessment (Nac, PFC, VTA and SN mRNA and protein levels for GDNF, BDNF and NGF)	Rats (P60), strain: Wistar	- Ibogaine ↑ GDNF mRNA in VTA and SN 24 h after injection - Ibogaine ↑ BDNF mRNA in all areas 24 h after injection △ - Ibogaine ↓ BDNF mRNA in PFC 3 h after injection △ - Ibogaine ↑ NGF mRNA in all areas 24 h after injection △ - Ibogaine ↑ NGF mRNA in PFC and VTA 24 h after injection △ - Ibogaine ↑ GDNF protein levels in VTA 24 h after injection - Ibogaine ↑ proBDNF protein levels in Nac 24 h after injection △	(Marton et al. 2019)
Behavioral assessment (OF, FST, EPM) Physiological assessment (5-HT levels, TPH-2, Ca^2^ATPase and ETC-1 activity, 5-HT2C-R expression, Cell morphology)	Rats (P60), strain: Wistar	- MP ↑ 5-HT levels in Hipp and PFC in the CUS model - MP ↑ TPH-2 activity in Hipp in the CUS model - MP ↑ 5-HT2c-R expression in PFC on CUS and healthy animals - MP ↑ 5-HT2c-R expression in Hipp on CUS but not on healthy animals - MP ↑ Ca^2^ATPase activity in Hipp and PFC in the CUS model - MP rescues ETC-1 downregulation induced by CUS - MP ↓ anxiety levels and anhedonical phenotype in CUS animals - MP ↓ cell morphological anomalies induced by CUS in Hipp and PFC	(Wankhar et al. 2020)
Behavioral assessment (OF) Physiological assessment (BrdU + cells) Electrophysiological assessment (Active membrane properties)	Mice (both sexes adults), strain: 5-HT_2B_-KO and *wt* control	- BW723C86 ↑ firing rate on pet1 + DRN ex vivo - BW723C86 prevent 5-HT1A agonist induced inhibition of pet1 + DRN in vivo - MDMA fail to ↑ locomotor activity on 5-HT2B-KO - Fluoxetine fails to ↑ BrdU + cells in 5-HT2B-KO ☆	(Belmer et al. 2018)
Behavioral assessment (TFC) Physiological assessment (BrdU + cells)	Mice (adult males), strain: C57BL6	- Psilocybin do not change TFC score for acquisition; - Psilocybin ↑ fear response on trial 1 of TFC for extinction - Psylocibin ↓ fear response on trial 10 of TFC for extinction - Psylocibin ↓ BrdU + cells in DG ☆ - NBOMe ↓ BrdU + cells in DG ☆	(Catlow et al. 2013)
Physiological assessment (AA and monoamines levels, utilization rate of monoamines)	Rats (P60 males), strain: Wistar	- Ayahuasca ↑ GABA levels in Hipp - Ayahuasca ↓ Glycine and GABA levels on the amygdala - Ayahuasca ↑ 5-HT on the Hipp - Ayahuasca ↑ all monoamines on the amygdala - Ayahuasca ↓ 5-HT use rate on Hipp - Ayahuasca ↓ monoamine consumption on amygdala	(Castro-Neto et al. 2013)
Behavioral assessment (Task oriented behavior; HTR)	Mice (P120-P150), strain: C57BL6; Zebrafish larvae	- DMT synthetic isoforms have comparable effect to its natural counterparts in a cue-oriented locomotion protocol - 5-MeO-IsoDMT does not produce any HTR	(Dunlap et al. 2020)
Physiological assesment (BrdU + and DCX + cells) Behavioral asessment (MWM; NORT)	Mice (adult males), strain: C57BL6	- DMT ↑ survival and proliferation of BrdU + and DCX + cells, which is blocked by Sigma-1R antagonist ☆△ - DMT ↓ escape latency in MWM is blocked by 5-HT2AR antagonist - DMT ↑ exploration time in NORT	(Morales-Garcia et al. 2020)
**In vitro studies**			
Physiological assessment (cell viability by MTT and Calcein assay; ki67 + and PI + cells)	SH-SY5Y cells	- AYA1, CEB, CEP all doses ↑ cell viability with 48 h incubation - AYA2 ↑ cell viability with 72 h incubation	(Katchborian-Neto et al. 2020)
Physiological assessments (Neurite growth)	Cultured rat cortical neurons	- DMT, isoDMT and 1-Me-DMT ↑ neurite growth △ - 5-HT2A binding is necessary to neurite growth effect of all psychoplastogens tested	(Dunlap et al. 2020)
Physiological assessments (Neurite growth, Spinogenesis)	Cultured rat cortical neurons	- DOI, DMT, LSD ↑ neurite growth and spinogenesis △ - The effects of psychoplastogens are similar to increased BDNF in culture medium, and both act through TrkB and mTOR cascade as well 5-HT2A signaling △	(Ly et al. 2018)
Physiological assessment (Musashi1, Nestin, Sox-2, PCNA, Tuj, Map-2, GADPH, GFAP, CNPase levels)	Cultured mice SGZ neurons	- Chronic DMT ↓ of Musashi1, Nestin and SOX2 expression levels are blocked by Sigma-1R antagonist △ - Chronic DMT ↑ number of neurospheres and PCNA expression levels are blocked by Sigma-1R antagonist △ - Chronic DMT ↑ Tuj, Map-2, GFAP and CNPase are blocked by Sigma-1R antagonist	(Morales-Garcia et al. 2020)

☆ represents outcomes assessing cell proliferation through BrdU + labeling, and △ indicates outcomes assessing cellular processes related to proliferation, differentiation, or progenitor cell maintenance. Abbreviations: vDG (ventral dentate Gyrus); sEPSC (spontaneous excitatory post synaptic current); pyr (pyramidal neurons); Nac (Nucleus Accumbens); PFC (Prefrontal Cortex); SN (Substantia Nigra); Hipp (Hippocampus); VTA (Ventral Tegmental Area); CUS (chronic unpredictable stress model); OF (Open Field); TST (tail suspension test); FST (forced swim test); EPM (elevated plus maze); 5-HT (serotonin); TPH-2 (Tryptophan hydroxylase 2); ETC-1 (mitochondrial electron transporter complex-I); DCX (doublecortin); GC (granule cells); BrdU (Bromodeoxyuridine); AHP (after hyperpolarization); AP (action potential); LFP (local field potential); TFC (Trace Fear Conditioning); AA (amino acids) HTR (Head-Twitch response); GDNF (glial cell derived neurotrophic factor); BDNF (brain derived neurotrophic factor); NGF (nerve growth factor); pet1 [pheochromocytoma 12 ETS (E26 transformation-specific)]; DRN (dorsal Raphe Nucleus); MTT (3-[4,5-Dimethylthiazol-2-yl]− 2,5-diphenyl tetrazolium bromide); Ki67 (marker of Proliferation Ki-67); AYA1 (santo Daime’s Ayahuasca decoction); CEB (*Banisteriopsis caapi* crude extract); CEP (*Psychotria viridis* crude extract); AYA2 (Ayahuasca prepared by authors); TrkB (tropomyosin receptor kinase B); mTOR (mechanistic target of rapamycin); MP (methylpsilocin)

### Entactogens/empathogens

As atypical psychedelics (Calvey and Howells 2018), those compounds typically induce an emotional sense of “oneness”, notably enhancing empathy on top of the usual psychedelic experience (Nichols 1986). These unique properties distinguish them from other classes of drugs. All entactogens bind to 5-HT_1A_ and 5-HT_2_ receptors with varying affinities. Some phenethylamines, such as MDMA (3,4-methylenedioxymethamphetamine) and its analogs, affect other monoamines by blocking their uptake and forcing their release into the synaptic cleft (Papaseit et al. 2020).

Although some tryptamines are included as entactogens (Nagai et al. 2007) our review found only articles exploring the effects of MDMA within our scope. MDMA was initially developed in 1912 by Merck chemist Anton Köllisch to be an appetite suppressant, but it was never sold for this purpose (Kalant 2001). In the 1970s, psychopharmacologist Alexander Shulgin synthesized it and, after self-experimentation, suggested its use as a medicine to aid patients undergoing therapy for PTSD and addiction disorders (Shulgin and Shulgin 1991). In the 1980s, MDMA became a street drug, often sold illicitly as “molly” or “ecstasy” (NIDA 2024). Today, MDMA is in the final stages of clinical research for approval as a psychotherapeutic tool to treat PTSD (Emerson et al. 2014). The included articles explore the effects of MDMA during embryonic and/or neonatal stages (57.14%), adolescence (28.57%), and adulthood (28.57%) in murine models, with some articles examining more than one ontogenetic window (Supp. Table 5).

The findings of these studies, as summarized in Table 5, illustrate that the effects of MDMA on neurogenesis can vary depending on the dosage and duration of administration. In a study by Hernández-Rabaza et al., researchers attempted to mimic the binge consumption of MDMA often seen in social settings such as raves. They administered a dose of 40 mg/kg over two days to male rats (the age of the rats was not reported). This treatment did not affect the proliferation of new cells. However, when assessed 14 days after the treatment, there was a noticeable 40% decrease in new cells (BrdU + cells) in the dentate gyrus compared to the control group. The researchers also examined potential abnormalities in the growth of new neurons but found no difference between the MDMA-treated and control groups (Hernández-Rabaza et al. 2006). Moreover, a study by Catlow et al. investigated the effects of a longer, chronic administration of MDMA. Rats were given a lower dose of 5 mg/kg per day for 10 days. The researchers observed an initial increase in new cells (BrdU + cells) immediately after the MDMA treatment, but a significant decrease (around 40–50%) was noted 14 days later. The survival of these new neurons, measured by co-labeling BrdU and NeuN, was also reduced by approximately 40–50% (Catlow et al. 2010).

The results presented by Catlow et al. (2010) and Hernández-Rabaza et al. (2006) prompt further investigation into potential differential effects of MDMA across developmental stages. García & García (2016) report that a 60 mg/kg dose of MDMA (3 × 5 mg/kg over 4 days) more significantly impacted proliferation in adult rats (P48-P58) compared to adolescent rats (P27-P37), as demonstrated by ki67 and BrdU labeling (García-Cabrerizo and García-Fuster 2016). Given the unreported age of the rats in Hernández-Rabaza et al.‘s (2006) study, a direct comparison with García & García’s findings is not recommended. However, the reported age range of rats in Catlow et al.‘s study (P28-39) corresponds to adolescence/young adulthood, introducing an element of ambiguity.

Further, García & García’s study also revealed a positive correlation between mature BDNF (mBDNF) levels and numbers of BrdU + cells (*r* = 0.492), with a stronger correlation observed between 5-HT2CR expression and mBDNF (*r* = 0.713). This led the authors to posit that chronic MDMA exposure (60 mg/kg) negatively affects overall plasticity, with more pronounced effects in adult animals. Interestingly, no significant impact was found when a lower dosage of 15 mg/kg (3 × 5 mg within 1 day) was administered (García-Cabrerizo and García-Fuster 2016).

With the growing popularity and widespread use of MDMA among young people and adults (Armenian and Rodda 2020), three other studies focused on examining the effects of MDMA during early stages of life, given concerns about the drug’s potential teratogenic properties. In particular, Cho and collaborators investigated the impact on adult neurogenesis in the offspring from dams exposed to 1.25 or 20 mg/kg MDMA for 36 days (E6-P21) via oral gavage. At 11 weeks post-exposure, animals were treated with BrdU. The study found that both doses led to reduced cell proliferation when measured 24 h post-BrdU labeling post, but the higher dose of 20 mg/kg resulted in fewer BrdU-positive cells at 28 days post-BrdU labeling. Notably, the authors performed BrdU + NeuN + cell co-labeling assessment at 28 days examine the phenotype of surviving newly generated cells and validate their model; however, only qualitative images are provided without associated quantitative data. Moreover, only comparisons with female offspring in the experimental groups were reported in the assessment of the survival of precursor cells at 28 days (Cho et al. 2008).

In a teratology study conducted by Thompson et al. (2012) found that maternal ingestion of MDMA at a similar dose during E14-E20 increased fiber density in the prelimbic cortex (PLC) and CA1. Meanwhile, the expression of the norepinephrine transporter (NET) was elevated in the cornus ammonis regions, but not in the dentate gyrus (DG), Locus Coeruleus (LC), or PLC (Thompson et al. 2012). The authors suggested further exploration of NET upregulation to strengthen the association between downstream morphological (increased plasticity) and neurochemical abnormalities (upregulation of NE in the PFC and Nac) with previously reported cognitive and behavioral changes related to novelty (Koprich et al. 2003; Thompson et al. 2009, 2012) However, the authors emphasized that the existing data does not suffice to determine causality. Subsequently, Canales and Ferrer-Donato (2014) demonstrated that a dose of 10 mg/kg MDMA administered during a shorter pregnancy window (3 days, E13-E15) combined with alcohol significantly reduced neurogenesis (both proliferation and survival) by approximately 40% in adult female offspring compared to saline. Behavioral assessment via the Radial Arm Maze (RM) revealed about a 30% reduction in locomotion and a roughly 50% increase in working memory errors. Notably, the within-group variance was quite narrow, and only the group exposed to both drugs exhibited these impairments (Canales and Ferrer-Donato 2014).

We conclude that abuse of MDMA with high acute doses results in enduring imbalances in the monoamine system and subsequent downregulation of neurogenesis. Conversely, low doses have no detectable effects on morphological abnormalities in adult brain tissue. It is noteworthy that the phase 3 studies currently being conducted by the Multidisciplinary Association for Psychedelic Studies (MAPS) are utilizing doses ranging from 0.08 to 0.18 g of MDMA per patient (Mithoefer et al. 2019). Therefore, neurobiological studies should futher examine how low doses of MDMA impacts brain physiology.

**Table 5:** Results reported by the reviewed studies on Entactogens

Outcome variable	Subjects	Reported Results	Reference
**In vivo studies**			
Physiological assessment (BrdU + cells)	Mice (E6-P21), strain: C57BL6	- Embryonic MDMA exposure ↓BrdU + cells in adulthood ☆	(Cho et al. 2008)
Behavioral assessment (SC, CWM, MWM) Physiological assessment (BW)	Rats (P11-P20), strain: Sprague Dawley	- MDMA ↑ score in CWM - MDMA ↑ Path length, Heading error in MWM - MDMA ↓ quad accuracy and crossovers in MWM - MDMA ↓ BW	(Schaefer et al. 2013)
Physiological assessment (BRDU+, DCX+, KI67 + cells, neurite growth)	Rats (adult males), strain: Wistar	- MDMA has no effect on BrdU+, Ki67 + cells and neurite growth ☆△ - MDMA ↓ DCX + cells ☆△	(Hernández-Rabaza et al. 2006)
Behavioral assessment (CPP) Physiological assessment (BrdU+, NeuN + cells)	Rats (P28-P39), strain: Sprague Dawley	- MDMA ↑ CPP only at 2.5 mg/kg dose - MDMA ↑ BrdU + cells 24 h after BrdU injection ☆ - MDMA ↓ BrdU + cells 14 days after BrdU injection ☆ - MDMA ↓ co-labelled BrdU + and NeuN + cells ○	(Catlow et al. 2010)
Physiological assessment (Fiber density on PLC and Hipp, NET density, NE levels)	Rats (E14-P21), strain: Sprague Dawley	- MDMA ↑ fiber density in PLC and CA1 - MDMA ↑ NET density in CA1. CA2 and CA3 but not on DG - MDMA ↑ NE levels on PFC and Nac	(Thompson et al. 2012)
Behavioral assessment (RM) Physiological assessment (BrdU+, DCX + cells)	Rats (E13-E15), strain: Long Evans	- ETOH/MDMA ↑ WM errors in RM - ETOH/MDMA ↓ Locomotor activity in RM - ETOH/MDMA ↓ BrdU + and DCX + cells ☆△	(Canales and Ferrer-Donato 2014)
Physiological assessment (BW, BrdU+, Ki-67, proBDNF and mBDNF and 5-HT2cR)	Rats (P27-P50), strain: Sprague Dawley	- MDMA has no effect on BW - Chronic MDMA ↓ Ki-67 + cells △ - Chronic MDMA ↓ BrdU + cells only in P42 mice ☆ - Chronic MDMA ↓ proBDNF; - Chronic MDMA ↓ mBDNF only in P21 mice - 5-HT2cR levels increase as mBDNF in P42 chronic MDMA treated mice	(García-Cabrerizo and García-Fuster 2016)

☆ represents outcomes assessing cell proliferation through BrdU + labeling, ○ represents assessing neurogenesis through BrdU + NeuN + cell co-labeling, and △ indicates outcomes assessing cellular processes related to proliferation, differentiation, or progenitor cell maintenance. Abbreviations: SC (straight Channel test); CWM (Cincinnati Water Maze); MWM (Morris Water Maze); BW (Body Weight); BrdU (Bromodeoxyuridine); DCX (Doublecortin); KI67 (marker of Proliferation Ki-67); CPP (conditioned place preference); PLC (pre-limbic cortex); Hipp (Hippocampus); NET (noradrenergic transporter); NE (Noradrenaline); DG (Dentate Gyrus); PFC (Prefrontal Cortex); Nac (Nucleus Accumbens); ETOH (ethanol); RM (Radial Maze); proBDNF (precursor protein of BDNF); mBDNF (mature BDNF)

### Other psychedelics

In this section, we go through additional articles that employed psychedelic drugs from chemical categories not previously discussed. These include the substituted amphetamine with psychedelic effects 2,5-Dimethoxy-4-iodoamphetamine (DOI) (Belmer et al. 2018; Berthoux et al. 2019; Jha et al. 2008; Jones et al. 2009; Ly et al. 2018; Marinova et al. 2017) and the substituted phenethylamine 25I-NBOMe (Table 6, Supp. Table 6) (Catlow et al. 2013).

A group from the French National Institute of Health and Medical Research (INSERM) assessed the capability of the 5-HT_2B_R to regulate the DRN serotonin release. Activation of pet1 + DRN cells by the tryptamine BW723C86 (1µM), a 5-HT_2B_ receptor agonist, administered ex vivo increased the spontaneous firing rate. Intriguingly, in subsequent experiments, 5 mg/kg DOI in vivo failed to boost the HTR in animals with a knockout of the 5-HT_2B_R, and 20 mg/kg MDMA in the same genotype also failed to replicate the increase in locomotor response observed in control mice. These behaviors were evaluated using the Open Field (OF) test (Belmer et al. 2018), indicating that 5-HT_2B_R may play a larger role in the behavioral effects of psychedelics on mice than previously believed.

Using BrdU labelling technique, it was reported that neither LSD (0.5 mg/kg) nor DOI (8 mg/kg) could increase proliferation in rats (age not reported) under either chronic (7 days) or acute treatment regimens (Jha et al. 2008). Another group from the Northwestern University Feinberg School of Medicine in Chicago, Illinois, reported that 1µM DOI could transiently promote dendritic remodeling in cultured cortical pyramidal neurons from rats, and this plasticity was mediated by Kalirin-7. The change involved the enlargement of spines, which began 30 min after exposure and returned to control size 60 min later (Jones et al. 2009). Ly and colleagues from UC Davis, in an article discussed previously (Table 2), extended the scope of their findings to other species, demonstrating that LSD and DOI could promote neuritogenesis in cultured neurons from mice and *Drosophila* larvae (Ly et al. 2018). Additionally, Marinova and colleagues employed human neuroblastoma SK-N-SH cells, TrkA is necessary for neurite extension (Marinova et al. 2017).

The only report found to investigate DOI using electrophysiological methods was conducted by Berthoux and colleagues. They used 1µM DOI on ex vivo slices from P14-P21 mice and reported that DOI induced Long-Term Depression (LTD) in mPFC layer 5 pyramidal neurons, as evidenced by whole-cell patch-clamp. The amplitude of the AMPA ESPC (excitatory postsynaptic current) coming from layer 1 Pyramidal stimuli was almost halved, and this decrease was mediated by 5-HT2AR since mice knocked out for 5-HT2AR did not show the same response. The internalization of AMPA receptors containing the GluA2 subunit through Protein kinase C (PKC) was necessary for DOI-induced LTD but not when LTD was replicated by a fast electrical pairing protocol (Berthoux et al. 2019). Moreover, in an article already discussed in the tryptamine section (Table 2), Catlow and colleagues used the substituted phenethylamine 25I-NBOMe in a single experiment to explore if 5-HT2AR activation affects proliferation of adult-born granule cells (abGC) in mice (age not reported). All tested doses (0.1, 0.3, and 1 mg/kg) reduced proliferation, but only the 1 mg/kg dose had a significant effect on the number of BrdU+/NeuN + cells compared to saline-treated mice (Catlow et al. 2013).

In summary, these studies suggest that the substituted amphetamine DOI and the substituted phenethylamine 25I-NBOMe may have a range of effects on neural plasticity and behavior. These effects appear to be modulated by different serotonin receptors, including 5-HT2BR and 5-HT2AR. More research is needed to further understand the molecular mechanisms and the functional implications of these changes. Also, the specificity of the effects of these substances on different stages of neurogenesis needs to be further investigated.

**Table 6:** Results reported by the reviewed studies on other psychedelics not covered by previous classifications

Outcome variable	Subjects	Reported Results	Reference
**In vivo studies**			
Electrophysiological assessment (Layer1 -↑ Layer 5 EPSC)	Mice (P14-P21), strain: 5-HT_2A_-KO and *w.t.* control	- DOI ↓ Layer 1 AMPA ESPC Amplitude in Layer 5 Pyr mPFC - mPFC layer 5 Pyr of 5-HT_2A_-KO mice do not react to DOI treatment - GluA2 subunit internalizations necessary for DOI-induced LTD in layer 5 Pyr mPFC - PKC/ 5-HT_2A_R inhibitors block DOI-induced LTD in layer 5 Pyr mPFC	(Berthoux et al. 2019)
Physiological assessment (BrdU + cells)	Rats (adult males), strain: Wistar	- DOI does not change BrdU + cells on DG ☆ - Acute Ketanserin ↓ BrdU + cells on DG ☆ - Chronic Ketanserin ↑ BrdU + cells on DG ☆	(Jha et al. 2008)
Behavioral assessment (OF) Physiological assessment (BRdU + cells) Electrophysiological assessment (Active membrane properties)	Mice (both sexes adults), strain: 5-HT_2B_-KO and *wt* control	- BW723C86 ↑ firing rate on pet1 + DRN ex vivo - BW723C86 prevent 5-HT_1A_ agonist-induced inhibition of pet1 + DRN in vivo - DOI fail to ↑ HTR in 5-HT2B-KO - Fluoxetine fails to ↑ BRdU + cells in 5-HT2B-KO ☆	(Belmer et al. 2018)
**In vitro studies**			
Physiological assessment (5-HT2AR, PSD95, Kalirin-7 and bassoon localization; MUPP1 activity; Dendritic spine remodeling; PAK phosphorylation levels)	Cultured rat cortical neurons	- DOI promotes spine shape remodelling without changing global spine density through PAK phosphorylation and subsequent Kalirin-7 activity △	(Jones et al. 2009)
Physiological assessment (TrkA tyr Phosphorylation levels at AA residue Tyr490 and Tyr785, Neurite Extension)	Human neuroblastoma SK-N-SH cells	- DOI all doses for 30–120 min ↑ TrkA phosphorylation in SK-N-SH cells △ - DOI all doses for 30–120 min ↑ TrkA phosphorylation at AA residue Tyr490 but not Tyr785△ - 5-HT2A and 5-HT2B block do not affect TrkA phosphorylation induced by DOI △ - Chronic DOI 1,5 µM ↑ neurite extension (can be blocked by TrkA inhibitor) △	(Marinova et al. 2017)

☆ represents outcomes assessing cell proliferation through BrdU + labeling, and △ indicates outcomes assessing cellular processes related to proliferation, differentiation, or progenitor cell maintenance. Abbreviations: EPSC (excitatory post synaptic current); pyr (pyramidal); mPFC (medial Prefrontal cortex); GluA2 (AMPA receptor subunit GluA2); LTD (long term depression); PKC (protein kinase C); BrdU (Bromodeoxyuridine); DG (Dentate Gyrus); PSD95 (post-synaptic density protein 95); bassoon (pre synaptic cytomatrix protein bassoon); MUPP1 (multiple PDZ protein-1); Cos-7 (fibroblast-like cell line from monkey kidney); PAK (P21-activated kinase); TrkA (tropomyosin receptor kinase A); AA (amino acid); tyr (tyrosine)

## Conclusions

This systematic review sought to reconcile the diverse outcomes observed in studies investigating the impact of psychedelics on neurogenesis. Additionally, this review has integrated studies examining related aspects of neuroplasticity, such as neurotrophic factor regulation and synaptic remodelling, regardless of the specific brain regions investigated, in recognition of the potential transferability of these findings.

Our study revealed a notable variability in results, likely influenced by factors such as dosage, age, treatment regimen, and model choice. In particular, evidence from murine models highlights a complex relationship between these variables for CB1 agonists, where cannabinoids could enhance brain plasticity processes in various protocols, yet were potentially harmful and neurogenesis-impairing in others. For instance, while some research reports a reduction in the proliferation and survival of new neurons, others observe enhanced connectivity. These findings emphasize the need to assess misuse patterns in human populations as cannabinoid treatments gain popularity. We believe future researchers should aim to uncover the mechanisms that make pre-clinical research comparable to human data, ultimately developing a universal model that can be adapted to specific cases such as adolescent misuse or chronic adult treatment.

Ketamine, the only NMDA antagonist currently recognized as a medical treatment, exhibits a dual profile in its effects on neurogenesis and neural plasticity. On one hand, it is celebrated for its rapid antidepressant properties and its capacity to promote synaptogenesis, neurite growth, and the formation of new neurons, particularly when administered in a single-dose paradigm. On the other hand, concerns arise with the use of high doses or exposure during neonatal stages, which have been linked to impairments in neurogenesis and long-term cognitive deficits. Some studies highlight ketamine-induced reductions in synapsin expression and mitochondrial damage, pointing to potential neurotoxic effects under certain conditions. Interestingly, metabolites like 2R,6R-hydroxynorketamine (2R,6R-HNK) may mediate the positive effects of ketamine without the associated dissociative side effects, enhancing synaptic plasticity and increasing levels of neurotrophic factors such as BDNF. However, research is still needed to evaluate its long-term effects on overall brain physiology. The studies discussed here have touched upon these issues, but further development is needed, particularly regarding the depressive phenotype, including subtypes of the disorder and potential drug interactions.

Harmala alkaloids, including harmine and harmaline, have demonstrated significant antidepressant effects in animal models by enhancing neurogenesis. These compounds increase levels of BDNF and promote the survival of newborn neurons in the hippocampus. Acting MAOIs, harmala alkaloids influence serotonin signaling in a manner akin to selective serotonin reuptake inhibitors SSRIs, potentially offering dynamic regulation of BDNF levels depending on physiological context. While their historical use and current research suggest promising therapeutic potential, concerns about long-term safety and side effects remain. Comparative studies with already marketed MAO inhibitors could pave the way for identifying safer analogs and understanding the full scope of their pharmacological profiles.

Psychoactive tryptamines, such as psilocybin, DMT, and ibogaine, have been shown to enhance neuroplasticity by promoting various aspects of neurogenesis, including the proliferation, migration, and differentiation of neurons. In low doses, these substances can facilitate fear extinction and yield improved behavioral outcomes in models of stress and depression. Their complex pharmacodynamics involve interactions with multiple neurotransmission systems, including serotonin, glutamate, dopamine, and sigma-1 receptors, contributing to a broad spectrum of effects. These compounds hold potential not only in alleviating symptoms of mood disorders but also in mitigating drug-seeking behavior. Current therapeutic development strategies focus on modifying these molecules to retain their neuroplastic benefits while minimizing hallucinogenic side effects, thereby improving patient accessibility and safety.

Entactogens like MDMA exhibit dose-dependent effects on neurogenesis. High doses are linked to decreased proliferation and survival of new neurons, potentially leading to neurotoxic outcomes. In contrast, low doses used in therapeutic contexts show minimal adverse effects on brain morphology. Developmentally, prenatal and neonatal exposure to MDMA can result in long-term impairments in neurogenesis and behavioral deficits. Adolescent exposure appears to affect neural proliferation more significantly in adults compared to younger subjects, suggesting lasting implications based on the timing of exposure. Clinically, MDMA is being explored as a treatment for post-traumatic stress disorder (PTSD) under controlled dosing regimens, highlighting its potential therapeutic benefits. However, recreational misuse involving higher doses poses substantial risks due to possible neurotoxic effects, which emphasizes the importance of careful dosing and monitoring in any application.

Lastly, substances like DOI and 25I-NBOMe have been shown to influence neural plasticity by inducing transient dendritic remodeling and modulating synaptic transmission. These effects are primarily mediated through serotonin receptors, notably 5-HT2A and 5-HT2B. Behavioral and electrophysiological studies reveal that activation of these receptors can alter serotonin release and elicit specific behavioral responses. For instance, DOI-induced long-term depression (LTD) in cortical neurons involves the internalization of AMPA receptors, affecting synaptic strength. At higher doses, some of these compounds have been observed to reduce the proliferation and survival of new neurons, indicating potential risks associated with dosage. Further research is essential to elucidate their impact on different stages of neurogenesis and to understand the underlying mechanisms that govern these effects.

Overall, the evidence indicates that psychedelics possess a significant capacity to enhance adult neurogenesis and neural plasticity. Substances like ketamine, harmala alkaloids, and certain psychoactive tryptamines have been shown to promote the proliferation, differentiation, and survival of neurons in the adult brain, often through the upregulation of neurotrophic factors such as BDNF. These positive effects are highly dependent on dosage, timing, and the specific compound used, with therapeutic doses administered during adulthood generally yielding beneficial outcomes. While high doses or exposure during critical developmental periods can lead to adverse effects, the controlled use of psychedelics holds promise for treating a variety of neurological and psychiatric disorders by harnessing their neurogenic potential.

## Past and future perspectives

### Brain plasticity

This review highlighted the potential benefits of psychedelics in terms of brain plasticity. Therapeutic dosages, whether administered acutely or chronically, have been shown to stimulate neurotrophic factor production, proliferation and survival of adult-born granule cells, and neuritogenesis. While the precise mechanisms underlying these effects remain to be fully elucidated, overwhelming evidence show the capacity of psychedelics to induce neuroplastic changes. Moving forward, rigorous preclinical and clinical trials are imperative to fully understand the mechanisms of action, optimize dosages and treatment regimens, and assess long-term risks and side effects. It is crucial to investigate the effects of these substances across different life stages and in relevant disease models such as depression, anxiety, and Alzheimer’s disease. Careful consideration of experimental parameters, including the age of subjects, treatment protocols, and timing of analyses, will be essential for uncovering the therapeutic potential of psychedelics while mitigating potential harms.

Furthermore, bridging the gap between laboratory research and clinical practice will require interdisciplinary collaboration among neuroscientists, clinicians, and policymakers. It is vital to expand psychedelic research to include broader international contributions, particularly in subfields currently dominated by a limited number of research groups worldwide, as evidence indicates that research concentrated within a small number of groups is more susceptible to methodological biases (Moulin and Amaral 2020). Moreover, developing standardized guidelines for psychedelic administration, including dosage, delivery methods, and therapeutic settings, is vital to ensure consistency and reproducibility across studies (Wallach et al. 2018). Advancements in the use of novel preclinical models, neuroimaging, and molecular techniques may also provide deeper insights into how psychedelics modulate neural circuits and promote neurogenesis, thereby informing the creation of more targeted and effective therapeutic interventions for neuropsychiatric disorders (de Vos et al. 2021; Grieco et al. 2022).

### Psychedelic treatment

Research with hallucinogens began in the 1960s when leading psychiatrists observed therapeutic potential in the compounds today referred to as psychedelics (Osmond 1957; Vollenweider and Kometer 2010). These psychotomimetic drugs were often, but not exclusively, serotoninergic agents (Belouin and Henningfield 2018; Sartori and Singewald 2019) and were central to the anti-war mentality in the “hippie movement”. This social movement brought much attention to the popular usage of these compounds, leading to the 1971 UN convention of psychotropic substances that classified psychedelics as class A drugs, enforcing maximum penalties for possession and use, including for research purposes (Ninnemann et al. 2012).

Despite the consensus that those initial studies have several shortcomings regarding scientific or statistical rigor (Vollenweider and Kometer 2010), they were the first to suggest the clinical use of these substances, which has been supported by recent data from both animal and human studies (Danforth et al. 2016; Nichols 2004; Sartori and Singewald 2019). Moreover, some psychedelics are currently used as treatment options for psychiatric disorders. For instance, ketamine is prescriptible to treat TRD in USA and Israel, with many other countries implementing this treatment (Mathai et al. 2020), while Australia is the first nation to legalize the psilocybin for mental health issues such as mood disorders (Graham 2023). Entactogen drugs such as the 3,4-Methyl​enedioxy​methamphetamine (MDMA), are in the last stages of clinical research and might be employed for the treatment of post-traumatic stress disorder (PTSD) with assisted psychotherapy (Emerson et al. 2014; Feduccia and Mithoefer 2018; Sessa 2017).

However, incorporation of those substances by healthcare systems poses significant challenges. For instance, the ayahuasca brew, which combines harmala alkaloids with psychoactive tryptamines and is becoming more broadly studied, has intense and prolonged intoxication effects. Despite its effectiveness, as shown by many studies reviewed here, its long duration and common side effects deter many potential applications. Thus, future research into psychoactive tryptamines as therapeutic tools should prioritize modifying the structure of these molecules, refining administration methods, and understanding drug interactions. This can be approached through two main strategies: (1) eliminating hallucinogenic properties, as demonstrated by Olson and collaborators, who are developing psychotropic drugs that maintain mental health benefits while minimizing subjective effects (Duman and Li 2012; Hesselgrave et al. 2021; Ly et al. 2018) and (2) reducing the duration of the psychedelic experience to enhance treatment readiness, lower costs, and increase patient accessibility. These strategies would enable the use of tryptamines without requiring patients to be under the supervision of healthcare professionals during the active period of the drug’s effects.

Moreover, syncretic practices in South America, along with others globally, are exploring intriguing treatment routes using these compounds (Labate and Cavnar 2014; Svobodny 2014). These groups administer the drugs in traditional contexts that integrate Amerindian rituals, Christianity, and (pseudo)scientific principles. Despite their obvious limitations, these settings may provide insights into the drug’s effects on individuals from diverse backgrounds, serving as a prototype for psychedelic-assisted psychotherapy. In this context, it is believed that the hallucinogenic properties of the drugs are not only beneficial but also necessary to help individuals confront their traumas and behaviors, reshaping their consciousness with the support of experienced staff. Notably, this approach has been strongly criticized due to a rise in fatal accidents (Hearn 2022; Holman 2010), as practitioners are increasingly unprepared to handle the mental health issues of individuals seeking their services.

As psychedelics edge closer to mainstream therapeutic use, we believe it is of utmost importance for mental health professionals to appreciate the role of set and setting in shaping the psychedelic experience (Hartogsohn 2017). Drug developers, too, should carefully evaluate contraindications and potential interactions, given the unique pharmacological profiles of these compounds and the relative lack of familiarity with them within the clinical psychiatric practice. It would be advisable that practitioners intending to work with psychedelics undergo supervised clinical training and achieve professional certification. Such practical educational approach based on experience is akin to the practices upheld by Amerindian traditions, and are shown to be beneficial for treatment outcomes (Desmarchelier et al. 1996; Labate and Cavnar 2014; Naranjo 1979; Svobodny 2014).

In summary, the rapidly evolving field of psychedelics in neuroscience is providing exciting opportunities for therapeutic intervention. However, it is crucial to explore this potential with due diligence, addressing the intricate balance of variables that contribute to the outcomes observed in pre-clinical models. The effects of psychedelics on neuroplasticity underline their potential benefits for various neuropsychiatric conditions, but also stress the need for thorough understanding and careful handling. Such considerations will ensure the safe and efficacious deployment of these powerful tools for neuroplasticity in the therapeutic setting.

## Electronic supplementary material

Below is the link to the electronic supplementary material.

Supplementary Material 1

## Data Availability

Data is provided within the manuscript tables or supplementary information files.
