# Supplementary material for: Effects of psychedelics on neurogenesis and broader neuroplasticity: a systematic review
Source: Mol Med. 2024 Dec 19;30:244. doi: 10.1186/s10020-024-01013-4 (PMC11657683; doi:10.1186/s10020-024-01013-4)
Supplement: Supplementary file 1 — Supplementary Material 1 [file 10020_2024_1013_MOESM1_ESM.pdf]

Supp Table 1 General methods used by reviewed CB1 agonists studies

Compound	Dose/Via	Frequency of treatment	Disease model	Animal model (# of subjects/group)	Type of neurogenesis	Reference
***In vivo* studies**						
**AEA**	AEA (2 µM); WIN 55, 212-2 (2 µM);	Acute (5-10 minutes)	No	Mice C56BL7, E13.5-E18.5, PN145-PN180 $CB1R^{f/f;\ Dlx5/6\text{-}Cre}$ mice (n= 5); Rat (n=ND)	Embryonic	(Berghuis et al., 2007)
**HU210**	HU210 (0.010, 0.050 mg/kg) IP	Chronic (10, 20 days)	AD(APP23/PS45)	Mice P28 and P63 (Total = 89)	Adult	(Chen et al., 2010)
**HU210**	HU210 (0.1 mg/kg) IP	Acute (twice daily); Chronic (10 days)	No	Rats male Long-Evans, Wistar, Fischer (n= 3-7)	Adult	(Jiang et al., 2005)
**HU210**	HU210 (0.025, 0.050, 0.100 mg/kg) IP	Chronic (11 days twice a day) Dose escalate in 3 age windows (0.025mg/kg in P35-P37; 0.050mg/kg in P38-P41; 0.100mg/kg in P42-P45)	No	Rats Sprague Dawley P28 (n= 5-6)	Adult	(Lee et al., 2014)
**M-AEA**	M-AEA (5mg/kg) IP	Chronic (4 days)	No	Rats Sprague-Dawley (n=4-8)	Adult	(Rueda et al., 2002)
**THC**	THC (3 mg/kg) IP	Acute	No	Mice CD-1 Female P35, P60, P90 (n=8)	Adult	(Leishman et al., 2018)
**THC**	THC (1, 10, 30 mg/kg) SC	Acute	No	Rats Male P4-P7, P20-P25, P90-P120 (n=4-9)	Developmental, Adult	(Downer et al., 2007)
**THC**	THC (2.5, 5, 10 mg/kg) Daily oral	Chronic (11 days twice a day) Dose escalate in 3 age windows (2.5mg/kg in P35-P37; 5mg/kg in P38-P41; 10mg/kg in P42-P45)	No	Rats Sprague-Dawley P28 (Total = 164)	Adult	(Rubino et al., 2008)
**THC**	THC (2.5, 5, 10 mg/kg) Daily oral	Chronic (11 days twice a day) Dose escalate in 3 age windows (2.5mg/kg in P35-P37.; 5mg/kg in P38-P41; 10mg/kg in P42-P45)	No	Rats Females Sprague-Dawley P28 (n=4-6)	Adult	(Realini et al., 2011)
**THC**	THC (6 mg/kg) IP	Chronic (15 days)	No	Rats Male Sprague-Dawley P28-42 (n= 10-11)	Adult	(Steel et al., 2014)
**THC**	THC (0.75, 1.5, 3 mg/kg) IP	Acute (for 7 days)<br>Chronic (21 days)	No	Rats Male Sprague-Dawley P28 (n=6)	Adult	(Suliman et al., 2018)

**THC**	THC (2.5, 5, 10 mg/kg) Daily oral	Chronic (11 days twice a day) Dose escalate in 3 age windows (2.5mg/kg in P35-P37; 5mg/kg in P38-P41; 10mg/kg in P42-P45)	No	Rats Female Sprague-Dawley P28 (n=4-8)	Adult	(Cuccurazzu et al., 2018)
**THC; Win 55212-2**	THC (1,3, 10, 30 mg/kg) IP;<br>20 to 80 mg/kg daily oral.	Acute; Repeated; Chronic (21 days)	No	Mice C57BL6 Male (n=4-9)	Adult	(Kochman et al., 2006)
**THC**	THC (1, 5 mg/kg) s.c.	Chronic (12 and 24 days)	No	Male Mice P5-P35 (n = 3-8)	Developmental, Adult	(Beiersdorf et al., 2020)
**THC**	THC (0.3, 1, 3 mg/kg) i.p.	Chronic (escalating 0.3 mg/kg P35-37 -1 mg/kg P38-41, 3 mg/kg P42-45)	No	Male Rats (n =7-12 behavioral experiments; n = 5 IHC experiments)	Adult	(Poulia et al., 2021)
***In vitro* studies**						
**AEA**	AEA (5 µM)	Acute (24h exposure)	No	Rats Cultured Cortical Neurons (n = 3 cultures); HNSC.100 (n = 3 cultures); PC12 cells (n = 3 cultures)	N/A	(Rueda et al., 2002)
**AEA; THC**	THC (1µM) ; AEA (1-10 µM) *	Chronic (21 days) *	No	hCBiPSC (n= 3-5 cultures) for Electrophysiological experiments (n= 2-35 cells)	N/A	(Stanslowsky et al., 2017)
**AEA; Win 55,212-2**	AEA (100 µM); Win55,212-2 (20 µM)	Acute (15-30 minutes)	No	Cultured GABAergic neurons from Mice E18.5 expressing CB1R (N = 13) ; Xenopus laevis (N = 8-210)	Embryonic	(Berghuis et al., 2007)
**HU210**	HU210 (1, 10, 100nM, 1, 10 µM)	Acute (48hrs exposition) Chronic (8 days)	No	Cultured Hippocampal NS/PCs cells derived from E17 (n= 4-6 cultures)	Embryonic	(Jiang et al., 2005)
**HU210**	HU210 (0.01, 0.1, 1. 10, 100 nM)	Acute (10-15 minutes exposure)	No	Mouse Neuroblastoma N1E-115 cells (n = 3 cultures)	N/A	(Zhou and Song, 2001)
**HU210**	HU210 (0.03, 0.3,3 µM)	Acute (6 days)	Hyperglycemic neuropathy	PC12 cells (n = 4-6 cultures; 136-218 cells)	N/A	(Zhang et al., 2009)

**O-2545**	O-2545 (0.035,0.07, 0.35mg/ml)	Acute (10µl solution during gastrulation above the ventral side of embryos)	No	Chicken embryos S3⁺-E8⁻, Charles River lab (n=3-54)	Embryonic	(Psychoyos et al., 2008)
**THC**	THC (5nM - 50µM)	Acute (2h)	No	Rat Cultured Cortical Neurons (n = 5-6 cultures)	N/A	(Downer et al., 2007)
**THC**	THC (3 µM); 2-AG (1 µM)	Acute (15-30 minutes and 24 hours)	No	hiPSC (n= 3 cultures per hiPSC line)	N/A	(Shum et al., 2020)
**THC**	THC (100 nM, 1, 7.5, 10 µM)	Acute (24 hours)	No	mice E14.5 cortex culture (n =15-33)	N/A	(Beiersdorf et al., 2020)
**Human studies**						
**THC; CBD**	THC/CBD ratio of hair samples and self-report of consumption	N/A	No	Healthy cannabis users and IQ, age matched controls (N=11-13)	N/A	(Demirakca et al., 2011)

AD (Alzheimer's Disease); THC ((−)-trans-Δ9-tetrahydrocannabinol); N/A (Not Applicable); SC (Sub-Cutaneous); HNSC.100 (human neural stem cell line 100); PC12 (cell line derived from a pheochromocytoma of the rat adrenal medulla);* = complex dosage and time exposition due to elevated cytotoxicity on concentrations above 10 µM for both drugs; hCBiPSC (human cord blood-derived induced pluripotent stem cell); IQ (Intelligence quotient); NS/PCs (Neural Stem/Progenitor Cells); S# (# of somites in the embryo).

Supp Table 2. General methods used by reviewed NMDA antagonists' studies

Psychedelic Used	Dose/via	Frequency of treatment	Disease model	Animal model (# of subjects/group)	Type of Neurogenesis	Reference
			***In vivo* Studies**			
**Ketamine**	Ketamine (30mg/kg) IP	Acute (3 days at P7, P9, P11)	No	Mice Male C57BL6 P11 and P70 (n = 4-7)	Developmental	(Schiavone et al., 2020)
**Ketamine**	Ketamine (30mg/kg) IP	Chronic (14 days)	Alcohol co-administration	Rats Male Sprague Dawley P35-42 (n=3-8)	Adult	(Zuo et al., 2018)
**Ketamine**	Ketamine (1, 10 mg/kg); Ketamine/lithium (1mg/10mg/kg) IP	Acute	No	Rats Male Sprague-Dawley (n= 4-8 animals; 8-17 cells)	Adult	(Liu et al., 2013)
**Ketamine**	Ketamine (30 mg/kg) IP	Chronic (5 days)	No	Rats Male Sprague-Dawley P56 (n = ND)	Adult	(Keilhoff et al., 2004)
**Ketamine**	Ketamine (4, 8, 20 mg/kg) IP	Acute	No	Rats Male Sprague-Dawley P70-P95 (n = 7-14)	Adult	(Goulart et al., 2010)
**Ketamine**	Ketamine (5, 10, 80 mg/kg) IP	Acute	No	Rats Male Sprague-Dawley (n =4-8)	Adult	(Li et al., 2010)
**Ketamine**	Ketamine (0.25, 0.5 mg/kg); IP	Chronic (11 days)	No	Rats Male Wistar (n=8)	Adult	(Akinfiresoye and Tizabi, 2013)
**Ketamine**	Ketamine (10 mg/kg) IP	Acute	No	Rats Male Sprague Dawley (n = 6-8)	Adult	(Lepack et al., 2015)
**Ketamine**	Ketamine (40 mg/kg) IP	Acute (4 times with 1h inter-dose interval)	No	Rats P7 (n = 5-6)	Developmental	(Huang et al., 2016)
**Ketamine**	Ketamine (10 mg/kg) IP	Acute	No	Rats Females Sprague-Dawley P56 (n = 3 animals; 11-42 neurons	Adult	(Ly et al., 2018)
**PCP**	PCP (10 mg/kg/day) SC	Chronic (12 days from E6-E18)	No	Mice CD-1 P1/P7/P56 (n=5-20)	Developmental	(Toriumi et al., 2012)
**PCP**	PCP (7.5 mg/kg) IP	Acute; Chronic (5 and 14 days); Chronic with challenging Dose on day 21	No	Rats Male Sprague-Dawley (n= 6)	Adult	(Liu et al., 2006)
**PCP**	PCP (5 mg/kg) SC	Chronic (14 days E8-E21)	No	Rats Sprague-Dawley P21 (n=3-8)	Embryonic	(Tanimura et al., 2009)

**PCP**	PCP (1-10 mg/kg) IP	Chronic (14 days)	No	Mice Male CD-1 P56 (n = 6-7)	Adult	(Maeda et al., 2007)
			***In vitro* Studies**			
**PCP**	PCP (1 µM)	Acute (24 hours)	No	Primary PFC mice cultured neurons (n = 3)	Developmental	(Zhang et al., 2016)
			**Human Studies**			
**Ketamine**	Ketamine dependence (PANSS questionnaire)	N/A	Hospitalized addicted subjects	Ketamine addicted (n= 155) and matching healthy control (n=80)	N/A	(Fan et al., 2015)
**Ketamine**	0.71 mg/kg via ND	Acute	Cocaine non-treatment seeking addicted voluntaries	Cocaine addicted (n=20)	N/A	(Dakwar et al., 2018)
**Ketamine**	0.5mg/kg IV	Acute (7 days after discontinuation of standard medication)	Bipolar patients within a depression episode and resistant to classical antidepressants	Treatment resistant depressive patients (n = 25)	N/A	(Rybakowski et al., 2013)
**Ketamine**	0.5mg/kg IV	Acute	Treatment resistant MDD	Treatment resistant MDD (n = 30)	N/A	(Duncan et al., 2013)
**Ketamine**	0.5mg/kg IV	Acute	Treatment resistant MDD	Treatment resistant MDD (n=22)	N/A	(Haile et al., 2014)

ND (Not Described); IP (Intraperitoneal); IV (Intravenous); SC (Subcutaneous); N/A (Not Applicable); P# (Post-Natal Day #); E# (Embryonic day #); MDD (Major Depressive Disorder); PFC (Pre-Frontal Cortex); PANSS (Positive and Negative Syndrome Scale).

Supp Table 3. General methods used by reviewed Harmala alkaloids studies

Psychedelic used	Dose/Via	Frequency of treatment	Disease model	Animal model (# of subjects/group)	Type of neurogenesis	Reference
			***In vivo* studies**			
**Harmine**	Harmine (5, 10, 15 mg/kg) IP	Acute	No	Rats Male Wistar P60 (n=15)	Adult	(Fortunato et al., 2009)
**Harmine**	Harmine (5, 10, 15 mg/kg) IP	Chronic (14 days)	No	Rats Male Wistar P60 (n=15)	Adult	(Fortunato et al., 2010a)
**Harmine**	Harmine (15mg/kg) IP	Chronic (7 days)	Chronic Mild Stress	Rats Male Wistar P60 (n=15)	Adult	(Fortunato et al., 2010b)
**Harmine**	Harmine (10, 20 mg/kg) IP	Chronic (10 days)	Unpredictable Chronic Stress	Mice C57BL6 Male P56-P70 (n=10)	Adult	(Liu et al., 2017)
			***In vitro* studies**			
**Harmine**	Harmine (7.5 µM)	Not reported	No	hNPCs (n = 6)	N/A	(Dakic et al., 2016b)
**Harmine; Tetrahydroharmine; Harmaline; Harmol**	Harmine (1 µM); T-Harmine (1 µM); Harmaline (1 µM); Harmol (1 µM)	Chronic (7 days); Acute (3 days)	No	Neurospheres from adult mice SGZ and SVZ (n= 3 independent cell cultures)	Adult	(Morales-García et al., 2017)

SVZ (Subventricular Zone); SGZ (Subgranular Zone); IP (Intraperitoneal); hNPCS (Human neural progenitor cells).

Supp Table 4 General methods used by reviewed Psychoactive tryptamines studies

Psychedelic used	Dose/Via	Frequency of treatment	Disease model	Animal model (# of subjects/group)	Type of neurogenesis	Reference
			***In vivo* studies**			
**DMT; LSD; DOI; Ketamine.**	Drosophila experiments, DOI, LSD (ND);<br>DMT 1, 10 mg/kg<br>Ketamine 10 mg/kg	Acute	No	Drosophila (n= ND); Rats Females Sprague-Dawley P56 (n = 3 animals; 11-42 neurons)	Adult	(Ly et al., 2018)
**5-MeO-DMT**	5-MeO-DMT (100 µg) ICV	Acute	No	Mice C56BL7 P55-P70 (n =3-6)	Adult	(Lima da Cruz et al., 2018)
**5-MeO-DMT**	5-MeO-DMT (20mg/kg) IP.	Acute	Tinnitus (acute salicylate model)	Mice C56BL7 P120-P150 (N=8)	Adult	(Winne et al., 2020)
**Ibogaine**	Ibogaine (20, 40 mg/kg) IP.	Acute	No	Rats Male Wistar P (n = 6)	Adult	(Marton et al., 2019)
**Methylpsilocin**	Methylpsilocin (0.7 mg/kg) IP	Acute and sub-acute (7 days)	CUS	Rats Male Wistar (N=6)	Adult	(Wankhar et al., 2020)
**BW723C86; MDMA, DOI,**	BW723C86 (1µm); MDMA (20 mg/kg) IP.; DOI (1,3,5mg/kg) IP.	Acute	5-HT2B KO; WT	Mice Male mixed B6::129S2 P21-28 (n= 3-5)	Adult	(Belmer et al., 2018)
**Psilocybin; NBOMe**	Psilocybin (0.1,0.5,1, 1.5 mg/kg) IP.; NBOMe (0.1,0.3,1 mg/kg) IP	Acute	No	Mice male C57BL6 (n=6)	Adult	(Catlow et al., 2013)
**Ayahuasca**	Ayahuasca (250, 500, 800 mg/kg) oral	Acute	No	Rats Male Wistar (n= 5-8)	Adult	(Castro-Neto et al., 2013)
**DMT and many isoforms**	mice = DMT (5ml/kg*) IP.; Zebrafish = DMT (28µM-200µM)	Acute	No	Mice both genders C57BL6 P (N= 3-8); Zebrafish larvae (n = 8 larvae per well, 18 wells per conditions	Adult/Developmental	(Dunlap et al., 2020)
**DMT**	DMT (2mg/kg) i.p.	Acute (4 days); Chronic (every other day for 21 days)	No	Adult Male Mice P90 (n = 5)	Adult	(Morales-Garcia et al., 2020)

			***In vitro* studies**			
**Ayahuasca**	all compounds tested at 1, 1.5, 2.5, 10.5 µg/mL	Acute (48h-72h incubation)	6-OHDA induced Neurodegeneration (Parkinson's Disease model)	SH-SY5Y cells (n= 3 cultures)	N/A	(Katchborian-Neto et al., 2020)
**DMT and many isoforms**	DMT (10µM -10 pM); IsoDMT (10µM -10 pM)	Acute (1h exposure)	No	Rats Cultured Cortical Neurons 6 days-in-vitro (n=46-79 neurons)	N/A	(Dunlap et al., 2020)
**DOI; DMT; LSD**	DOI (10 µM); DMT (90 µM); LSD (10 µM).	Acute (24h exposure)	No	Rats Cultured Cortical Neurons 19 days-in-vitro (n=46-79 neurons)	N/A	(Ly et al., 2018)
**DMT**	DMT (1µM)	Acute (24 hours); Chronic (7 days)	No	Culture mice SGZ cells (n =3 independent cell cultures)	N/A	(Morales-Garcia et al., 2020)

IP (intraperitoneal); ICV (intracerebroventricular); N/D (Not Declared); N/A (Not Applicable); CUS (Chronic Unpredictable Stress model); MP (methylpsilocin) SH-SY5Y (human cell line derived from neuroblastoma).

Supp Table 5 General methods used by Entactogens reviewed studies

Psychedelic used	Dose/Via	Frequency of treatment	Disease model	Animal model (# of subjects/group)	Type of neurogenesis	Reference
			**In vivo**			
**MDMA**	MDMA (1.25, 20mg/kg) Oral	Chronic (36 days)	No	Mice from embryo to adolescence (E6-P21) C57BL6 (n=16)	Embryonic/Adult	(Cho et al., 2008)
**MDMA**	MDMA (10mg/kg) IP.	Chronic (4x daily with a 2 h interdose interval by 10 days)	No	Rats Male Sprague Dawley P11-20 (n= 16-27)	Developmental	(Schaefer et al., 2013)
**MDMA**	MDMA (5.mg/kg) IP.	Acute (8 times with a 6h interdose interval)	No	Rats Male Wistar (n= 6-7)	Adult	(Hernández-Rabaza et al., 2006)
**MDMA**	MDMA (1.25, 2.5, 5 mg/kg) IP.	Chronic (10 days)	No	Rats Male Sprague-Dawley P28-39 (n=7-11)	Adult	(Catlow et al., 2010)
**MDMA**	MDMA (15mg/kg) Oral	Chronic E14-E20 (2x at day with an 8h interdose interval)	No	Rats Sprague-Dawley E14-P21 (n = 6)	Embryonic	(Thompson et al., 2012)
**MDMA**	MDMA (10mg/kg) Oral	Acute E13-E15 (2x at day with an 8h interdose interval)	No	Rats Long Evans E13-E15(n = 6)	Embryonic	(Canales and Ferrer-Donato, 2014)
**MDMA**	MDMA (5.mg/kg) IP.	Acute (3x at day with 2-3h interdose interval for 1 day) or<br>Chronic (3x at day with 2-3h interdose interval for 4 days)	No	Rats Male Sprague-Dawley P27-P29/P48-P50 (n= 18)	Adult	(García-Cabrerizo and García-Fuster, 2016)

IP (intraperitoneal).

Supp Table 6 General methods used by reviewed studies using other psychedelics.

Psychedelic used	Dose/Via	Frequency of treatment	Disease model	Animal model (# of subjects/group)	Type of neurogenesis	Reference
			***In vivo* studies**			
**DOI**	DOI (1µm)	Acute	5-sHT$_{2A}$R-KO	Mice "WT" and 5-HT$_{2A}$R-kO P14-P21 (n = ND, cells = 5-13)	Adult	(Berthoux et al., 2019)
**DOI; LSD**	DOI (8mg/kg); LSD (0.5mg/kg) IP.	Acute and chronic (7 days), same doses	No	Rats Male Wistar (n=4 - 7)	Adult	(Jha et al., 2008)
			***In vitro* studies**			
**DOI**	DOI (10, 100, 500 nM, 1 µM)	Acute (5-60 minutes)	No	Cultured rat cortical neuron (n = ND); Cos-7 cells (n=ND)	N/A	(Jones et al., 2009)
**DOI**	DOI (1, 2, 5 µM)	Acute (30min or 120 min exposure); Chronic (6 days exposure)	No	Human neuroblastoma SK-N-SH cells (n=3 independent cell cultures)	N/A	(Marinova et al., 2017)

WT (Wild Type); IP (Intraperitoneal); Cos-7 (); ND (Not described); N/A (Not Applicable).
